# Single-Cell Gene Expression Analysis Revealed Immune Cell Signatures of Delta COVID-19

**DOI:** 10.3390/cells11192950

**Published:** 2022-09-21

**Authors:** Abusaid M. Shaymardanov, Olga A. Antonova, Anastasia D. Sokol, Kseniia A. Deinichenko, Polina G. Kazakova, Mikhail M. Milovanov, Alexander V. Zakubansky, Alexandra I. Akinshina, Anastasia V. Tsypkina, Svetlana V. Romanova, Vladimir E. Muhin, Sergey I. Mitrofanov, Vladimir S. Yudin, Sergey M. Yudin, Antonida V. Makhotenko, Anton A. Keskinov, Sergey A. Kraevoy, Ekaterina A. Snigir, Dmitry V. Svetlichnyy, Veronika I. Skvortsova

**Affiliations:** 1 Centre for Strategic Planning and Management of Biomedical Health Risks of the Federal Medical-Biological Agency, 10 Bld., 1 Pogodinskaya Str., Moscow 119121, Russia; 2 The Federal Medical Biological Agency (FMBA of Russia), 30 Volokolamskoe sh., Moscow 125310, Russia

**Keywords:** single-cell, COVID-19, immune system, monocytes, transcriptome

## Abstract

The coronavirus disease 2019 (COVID-19) is accompanied by a cytokine storm with the release of many proinflammatory factors and development of respiratory syndrome. Several SARS-CoV-2 lineages have been identified, and the Delta variant (B.1.617), linked with high mortality risk, has become dominant in many countries. Understanding the immune responses associated with COVID-19 lineages may therefore aid the development of therapeutic and diagnostic strategies. Multiple single-cell gene expression studies revealed innate and adaptive immunological factors and pathways correlated with COVID-19 severity. Additional investigations covering host–pathogen response characteristics for infection caused by different lineages are required. Here, we performed single-cell transcriptome profiling of blood mononuclear cells from the individuals with different severity of the COVID-19 and virus lineages to uncover variant specific molecular factors associated with immunity. We identified significant changes in lymphoid and myeloid cells. Our study highlights that an abundant population of monocytes with specific gene expression signatures accompanies Delta lineage of SARS-CoV-2 and contributes to COVID-19 pathogenesis inferring immune components for targeted therapy.

## 1. Introduction

The COVID-19 pandemic that started in 2020 significantly changed many aspects of social life and raised an urgent necessity for immunological studies to decipher molecular and cellular mechanisms associated with the viral infection. Single-cell sequencing techniques are one of the best approaches to achieve a profound understanding of the essential molecular pathways governing immune response. The latest discoveries of transcriptomes [1,2,3,4,5] and chromatin accessibility [4,6] revealed severe alterations in the immune response depending on the disease severity. The landscape of immune changes involves shifts in the level of dendritic cells, monocytes, and T-cells [4,7,8]. Notably, a decrease in T-cells and plasmacytoid dendritic cells was coupled with disease severity and reduced interferon IFN-γ expression by lymphocytes [8,9]). In addition, monocytes in severe cases display an increase of the type I IFN inflammatory genes combined with the reduction of the IFNα levels [10] and elevated levels for a large number of cytokines (IL-10, IL-6, IL-11, TNF) for severe of COVID-19 cases [11]. Furthermore, monocytes and macrophages recruitment play an essential role for immune response, accompanied by the release of cytokines and activating adaptive T- and B-cells function associated with the destruction of lung cells [12,13,14]. Immunological mechanisms governing severe COVID-19 involve monocytes with proinflammatory or immunosuppressive gene signatures [6,15,16]. Furthermore, investigations of gene expression identified several transcription factors as driver regulators of cytokine production and represent potential drug targets for COVID-19 treatment [17,18] and disease severity markers.

Comprehensive studies of gene expression identified several transcription factors as driver regulators of cytokine production and represent candidates for drug targets for COVID-19 treatment and disease severity markers [17,18]. Currently, several SARS-CoV-2 variants with different virus characteristics, including transmissibility, pathogenicity, and antigenicity have been identified [19,20,21] and require deeper investigations of the immune changes in COVID-19 both on cellular and gene expression levels. The primary aim is a detailed description of cell state repertoire accompanying COVID-19 severity. To this end, the single-cell RNA-seq (scRNA-seq) approach is a perfect instrument to study gene expression and transcription factors (TFs) regulatory network changes to uncover mechanisms involved in the immune response.

To elucidate molecular and regulatory pathways engaged in anti-COVID-19 response, we profiled peripheral blood mononuclear cells (PBMC) of 40 individuals infected with Wuhan-like (Section 2) or Delta SARS-CoV-2 variants with scRNA-seq. We identified changes in the main immune cell types levels, both depending on the severity and virus variant. Our results revealed core changes in the monocytes that gain strong inflammatory gene signatures in COVID-19 cases caused by the Delta variant. We investigated gene regulatory networks and predicted main transcriptional regulators and their target genes that contribute to cytokine storms and may point to potentially effective treatment strategies. To our knowledge, this is the first high-resolution transcriptomic study of blood mononuclear immune cells for cases caused by the Delta variant of SARS-CoV-2.

## 2. Materials and Methods

### 2.1. Sample Collection

All patients with COVID-19 (confirmed with PCR test) were recruited in the Federal Clinical Center for High Medical Technologies of the Federal Medical Biological Agency from June 2020 to August 2021. Patients were divided into mild/moderate and severe/critical groups according to the World Health Organization (WHO) diagnosis guidance. Healthy individuals were recruited as volunteers and had negative PCR test for COVID-19. Informed consent was obtained from all subjects involved in the study. For the COVID-19-positive samples and healthy individuals, peripheral blood was collected in EDTA tubes and processed within 4 h of collection. PBMCs were isolated with a FicollR Paque Plus (Sigma Aldrich (Merck KGaA, Darmstadt, Germany)) solution according to standard density gradient centrifugation methods. The PBMCs were resuspended in freezing media (90% fetal bovine serum, 10% DMSO) and frozen in a −80 °C freezer.

### 2.2. Single-Cell Genes (RNA-Seq)

To study the 3′ gene expression of single cells, libraries were prepared using the Chromium Next GEM Single Cell 3′ Reagent Kits v3.1 protocol (10× Genomics, Pleasanton, CA, USA) and sequenced using the NovaSeq6000 and NextSeq2000 (Illumina, San Diego, CA, USA) platforms.

The research material was samples of suspensions of PBMC cells. Biomaterial quality control was carried out using a Countess II FL cell viability counter and analyzer (Thermo Fisher Scientific, Waltham, MA, USA). Cell suspensions conforming to the protocol requirements were used for sample preparation of libraries. In the prepared Master Mix, 31.8 µL per sample, the studied suspension of cells of 13.8 µL and water without nucleases of 29.5 µL were added. The mixture was stirred using an automatic dispenser 10 times and applied to a chip to generate an emulsion. Then gel particles and oil were applied to the chip. The loaded chip was processed at The Chromium Controller station. The resulting emulsion was incubated for 45 min at a temperature of 53 °C, then 5 min at 85 °C and stored at 4 °C, the temperature of the amplifier lid is 53 °C, the volume of the mixture is 125 µL. The emulsion was cleaned after incubation as follows: 125 mL of Recovery Agent was added to each sample, the tubes with the mixture were kept for 2 min and carefully turned over, after which 130 mL of the lower pink phase were briefly centrifuged and selected. After that, the prepared Dynabeads Cleanup Mix was added to each sample and kept at room temperature for 10 min; then, the magnetic particles were washed with 80% alcohol and cDNA fragments were eluted using an EB buffer. Then amplification was performed to obtain a full-sized cDNA. After cDNA amplification, magnetic particle purification was performed and quality control of the purified cDNA was carried out using an automated Tape Station 4200 (Agilent, Santa Clara, CA, USA) electrophoresis system with a set of D5000 (Agilent, Santa Clara, CA, USA) and a Qubit 4 fluorimeter (Thermo Fisher Scientific, Waltham, MA, USA). For cDNA fragmentation, 10 µL of the sample and a prepared Fragmentation Mix were used. After fragmentation, the samples were cleaned bilaterally on magnetic particles. Then, the adapters were ligated: 50 µL of the prepared Adapter Ligation Mix was added to 50 µL of the sample and incubated for 15 min at 20 °C. After ligation, purification was carried out on magnetic particles. The final stage of library preparation was indexing PCR. For it, a set of Chromium™ i7 Sample Index Plate (10× Genomics, Pleasanton, CA, USA) was used for one-way indexing. To 30 µL of the sample, 60 µL of the prepared Sample Index PCR Mix and 10 µL of the index were added. The number of cycles in the indexing PCR was 12 for samples with a cDNA concentration of 4–25 ng/µL. After indexing, the samples were subjected to double-sided cleaning on magnetic particles, and quality control of the resulting libraries was carried out using Tape Station 4200 with a set of D1000 (Agilent, Santa Clara, CA, USA) and a Qubit 4 fluorimeter. The finished libraries were sequenced with coverage of >200 million readings per sample.

### 2.3. Generating Single-Nucleus Gene Expression Matrices, QC, and Filtering

RNA-Seq data were demultiplexed using Cell Ranger (v6.0.1) “mkfastq”, aligned, and a matrix of counts was created. The transcripts were aligned to the reference human GRCh38 genome. The resulting expression matrices were processed individually in R (v.4.0.2) using Seurat (v.3.2.3). Filters were applied to keep nuclei with 200–2500 genes and less than 15% mitochondrial reads. Filtered gene–barcode matrices were normalized with the ‘NormalizeData’ function and the top 2000 variable genes were identified using the ‘vst’ method in ‘FindVariableFeatures’. Gene expression matrices were scaled and centered using the ‘ScaleData’ function. Next, we performed principal component analysis (PCA) and UMAP transformation using the first 30 principal components. UMAPs of individual samples were inspected before integration. After quality control and filtering, we generated scRNA-seq matrix of the peripheral blood mononuclear cells (PBMC) that was further combined with publicly available single-cell data [7].

### 2.4. Dataset Integration

We compared Seurat CCA [22] and Harmony [23] methods to find the best balance between data alignment and preserving biological heterogeneity. First, we calculated the Jaccard similarity between all clusters identified for both integration methods and found a high agreement between the two approaches (Figure S1). We used integration based on Seurat CCA for further analysis that provides a more biological separation of cell groups. PCA and UMAP dimension reduction were performed based on the top 30 principal components. Nearest-neighbor graphs using the top 30 dimensions of the PCA reduction were calculated and clustering was applied (resolution = 0.8). Harmony was run on the PCA matrix using default parameters with patient ID as the batch key. We filtered out 2 cell clusters suspected to be doublets and had low quality metrics.

### 2.5. Cell-Type Identification

The main cell types were identified by manual annotation. The ‘FindAllMarkers’ function was applied to identify positive markers for each cluster with a minimal fraction of 25% and a log-transformed fold change threshold of 0.25.

### 2.6. Module Scores for Gene Signatures

The ‘AddModuleScore’ function was applied to calculate the average expression levels of gene signatures on a single-cell level. Sets of complement, hypoxia, inflammation, interferon-alpha, interferon-gamma responses, Kras, and TNFA signaling via NFKB were obtained from GSEA (https://www.gsea-msigdb.org/ accessed on 4 September 2022) to calculate the module score for each cell type.

### 2.7. Differential Gene Expression

The Wilcoxon rank-sum test was used to identify differentially expressed genes between two groups of cells and the log-transformed fold change was set to 0.25. The parameter ‘min. pct’ was set to 0.25 to assure that genes were detected at a minimum fraction of 25% of cells in either of the populations. P values were adjusted using Bonferroni correction.

### 2.8. GO and KEGG Pathways Enrichment (GSEA)

We used the clusterprofiler package for enrichment analysis [24]. For GO and KEGG analysis for each cell type we used significantly upregulated differentially expressed genes (logFC > 1.5). We used GO and KEGG terms with *p*.adj < 0.05.

### 2.9. Transcription Factor Regulatory Network Analysis

Transcription factor regulatory network analysis was performed using SCENIC [25] workflow (version 1.1.2). The transcription factor database based on the GRCh38 genome was downloaded using RcisTarget (version 1.6.0). Gene regulatory networks were inferred using GENIE3 (version 1.6.0). Enriched transcription factor-binding motifs, predicted candidate target genes (regulons), and regulon activity were inferred using RcisTarget. The transcription regulatory network was visualized using Igraph (1.2.7) and the Fruchterman–Reingold layout.

### 2.10. Cell–Cell Communication Analysis

Cell–cell communication analysis was conducted using the CellChat [26] software (version 1.1.0) with default parameters. In the CellChat, probability value represents the quantitative strength of a given ligand–receptor interaction. Probability calculated based on the law of mass action relying on the average expression of the ligand in a cell group and receptor by another cell group. Circle plot was generated with netVisual_diffInteraction function with parameter top = 0.15. We used the rankNet function with parameter do.stat = TRUE to determine whether there is significant difference between two datasets. Output of this function is a stacked bar plot. Dot plot and chord diagrams were generated with functions netVisual_bubble and netVisual_chord_gene respectively with default parameters.

### 2.11. Machine Learning Classifier

Classification was performed using the XGBoost library (ver. 1.4.1) [27]. We obtained all ligand–receptor pairs from CellChatDB. Expression profiles for L-R pairs of Delta and severe/critical cells were used as predictors and severity as class label. We classified the whole Monocyte compartment treating Delta and Wuhan-like variants as class labels relying on normalised expression of ligand–receptor pairs. 10 k-fold cross-validation was used to estimate model performance. We calculated Shapley values using the SHAP for XGBoost (ver. 0.1.1) R library [28] to estimate contribution to classification for every gene. For further analysis and visualization, we used genes with mean Shapley value > 0.

### 2.12. SARS-CoV-2 Genotyping

Genotyping on 9 swab samples have been performed with Illumina COVIDSeq Test (Illumina, San Diego, CA, USA) according to standard protocol. For all 9 cases have been confirmed, Delta variants have been confirmed using the HAVoc pipeline [29].

## 3. Results

### 3.1. The Immune Landscape of Delta Variant SARS-CoV-2 Infection

**Figure 1:**
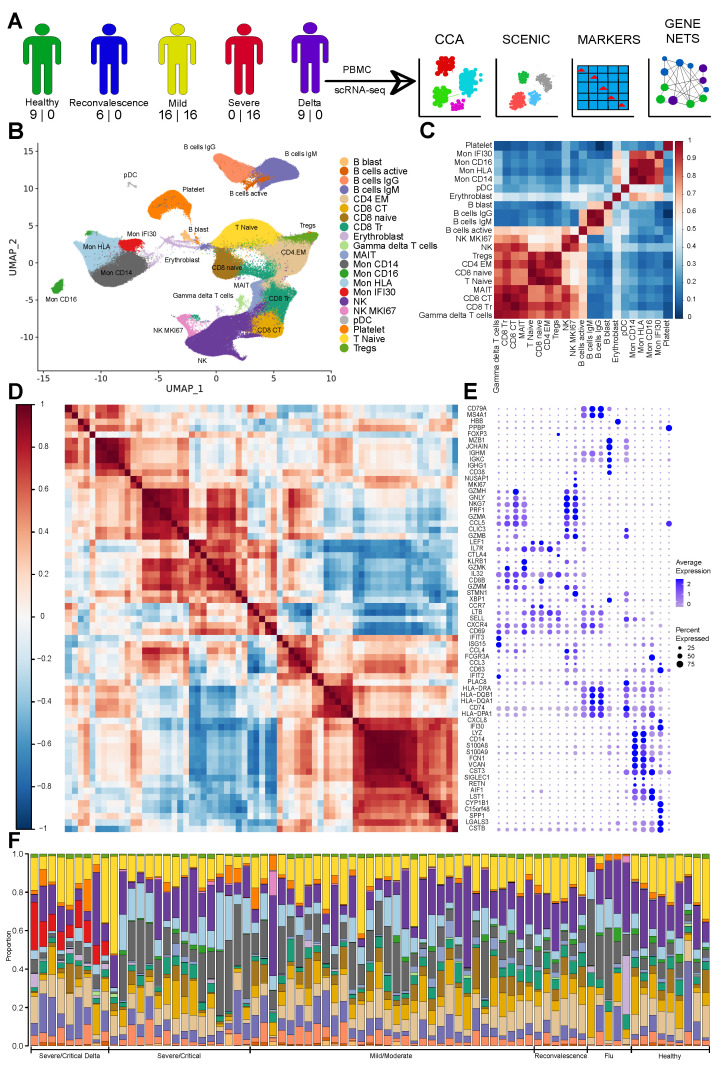
(**A**) Schematic diagram showing assays performed on the PBMC of COVID-19 patients with numbers of newly sequenced and publicly available samples (shown as number in corresponding order). The diagram shows performed computational analysis regarding computational data integration made with Seurat CCA and SCENIC, identification of marker genes, and computational reconstruction of the Gene Regulatory Network. (**B**) UMAP with identified cell types. (**C**) Cell–cell correlation heatmap based on Pearson correlation of the average expression of highly variable genes depicting across cell types similarity. (**D**) Gene expression Pearson correlation heatmap indicating formed gene modules by marker genes. (**E**) Dot plot with the expression of marker genes for each cell type ordered according to the correlation on the (**C**), and gene ordered based on (**D**). Three plots (**C**–**E**) allow joint view highlighting similarity based on activity level of marker genes and cell type closeness as follows from the average expression correlation of highly variable genes. (**F**) Stacked bar plot with cell type fractions for each sample colored according to the pallet of UMAP. We identified clearly visible Mon IFI30 cells fraction in the samples with Delta variant.

To investigate peripheral immune response specific for COVID-19 depending on the virus variant and severity of the symptoms, we generated scRNA-seq of the PBMC for 40 individuals (Table S1). We combined it with publicly available single-cell gene expression data [7] and analyzed individuals with COVID-19 (mild/moderate, severe/critical, convalescent), severe Flu, and healthy controls that never had COVID-19 infection (Section 2), yielding 76 datasets totally (Figure 1A). We sequenced 421,090 high quality cells across 40 samples, performed computational doublet removal, filtered cells using quality metrics, and integrated with public datasets (Figure 1A) that yielded 511,272 cells for further analysis.

Next, we performed computational data integration [22] followed by Louvain clustering to identify cell subtypes. Cells were annotated (Figure 1B) according to published marker genes with additional investigation using computational label transfer approach [30] (Figure S1). Clustering and unambiguity of marker gene expression yields 21 cell subtypes. Correlation based on the expression of highly variable genes indeed confirms their separation to distinct groups (Figure 1C). Gene-wise correlation of marker genes subset highlights certain coexpressed modules specific for each cell subtype (Figure 1D,E). In order to verify our clustering results obtained with Seurat CCA [22], we also performed integration with Harmony [23] (Figure S2A). We calculated the Jaccard similarity between all clusters identified with both integration methods and found a high agreement between the two approaches (Figure S2B). We used integration based on Seurat CCA for further analysis that provides a more biological separation of cell groups. Remarkably, the Treg cluster, a cell type easily distinguishable based on the expression of CD4, FOXP3, CTLA4, and TIGIT, was identified as a separate cluster of cells only with the CCA approach (Figure S2C,D).

We identified all main myeloid and lymphoid cell lineages (Figure 1B). For the lymphoid cell clusters, several states represented by CD4, CD8, mucosa-associated invariant T cells (MAIT), NK-cells have been found. The T naive cells are marked based on the CD8 or CD4 expression together with LEF1 and SELL [31]. Subtypes of the CD4, namely CD4 EM cells were found based on the high expression of the IL7R, and low SELL, LEF1. We detected 2 clusters of the CD8 cells - transitional CD8 (CD8 Tr) with high GZMK, GZMB, and cytotoxic (CD8 CT) with GZMH expression and high PRF1 (Figure 1D) [32]. Also, we revealed two NK subtypes—NK and NK MKI67 cells (Figure 1B–E and Figure S3), and both have high expression of the cytotoxic marker genes (GNLY, NKG7, GZMA, PRF1). Other studies identified depletion of the NK cells with increase of the symptoms severity which can be explained by their mobilisation and migration to different tissues or additional cell death caused by the virus [33]. Notably, previous studies identified an increase of the proliferating NK cells (high MKI67) with severity of COVID-19 and highlighted the role of IL-15 and TGF beta [34] in the cell state generation, thus emphasising involvement of NK subtypes in COVID-19 pathogenesis. We identified a small population of the pDC cells relying on the expression of CLEC9A, GZMB, IGKC, and JCHAIN [35]. Profound and detailed profiling of gene expression on a single-cell level allows characterization of subtypes that are highly important for understanding of the COVID-19 immune response.

We also abundantly detected various populations of the myeloid cells. First, we identified classical (CD14+) and CD16+ monocytes with high LYZ and MHCII (Figure S3). For the cohort of severe COVID-19 cases with Delta variant, we revealed high divergence of the monocyte populations with specific cell clusters that might be associated with pathogenesis, recovery, and immune response (Figure 1F). Surprisingly, we identified a subtype of cells (Mon IFI30) that topologically colocalized with monocytes on the UMAP plot (Figure 1B–E and Figure S3) and did not show high expression of the well-known monocyte receptors (CD14 or CD16). This cell state is characterized by proinflammatory expression profile (CTSB, CST3, and LGALS3), and high levels of CXCL8, IFI30, and C15orf48. It was shown that CXCL8 acts on CXCR1 and CXCR2 receptors [36] and leads to the elimination of pathogens but might be associated also with tissue injury, enhancing fibrosis, and guiding of neutrophils [37]. We also identified clusters of monocytes (Mon HLA) with a similar to classical monocytes gene activity profile but with high HLA-DPA1 expression (Figure 1E). Overall, investigation of the immune profiles with single-cell sequencing yields a profound description of the PBMC for patients and healthy individuals that provides a further rationale for possible treatment strategies.

### 3.2. Severe COVID-19 Caused by Delta Variant Associated with Shifts in Immune Cell Composition

**Figure 2:**
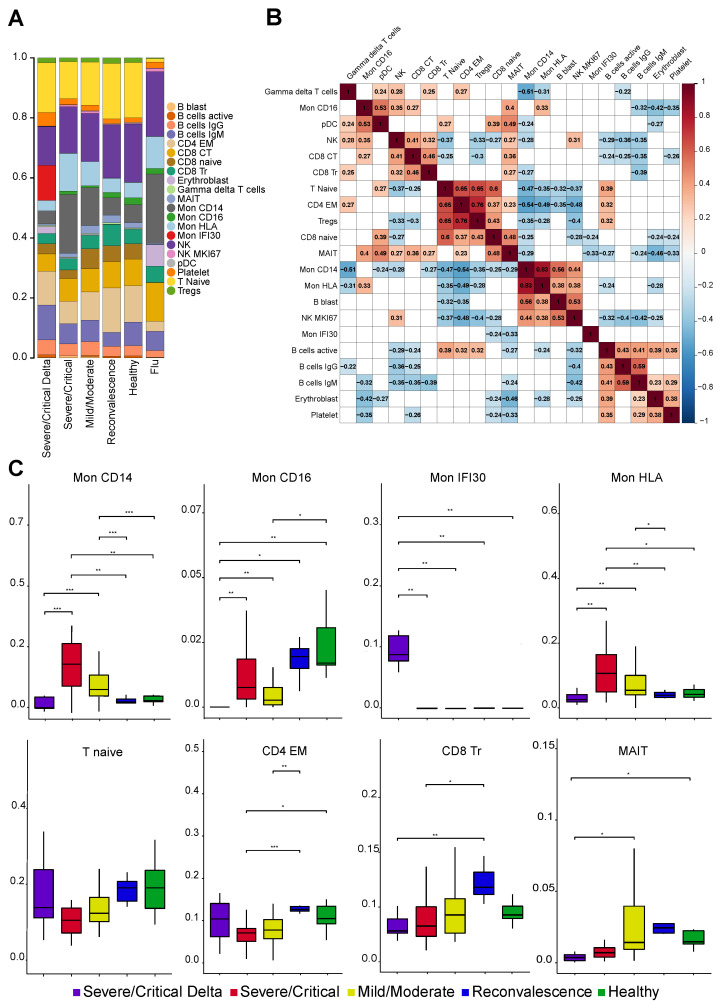
(**A**) Stacked bar plot with the average fraction of cell subtypes across samples. (**B**) Pearson correlation heatmap of the cell fraction for each subtype across samples. Statistically not significant (*p*-value > 0.05 according to cor.test in R) elements are blank. (**C**) Boxplot with the fraction of cell subtypes in each sample. *, **, *** means significant changes calculated with Wilcoxon rank-sum and shown as bar.

Host–pathogen immune responses during viral infections are usually associated with cellular and molecular shifts in the fractions of the immune cell (Figure 2A,B). To investigate the effect and immunological mechanisms of the COVID-19, we revealed pronounced changes of cell type composition depending on the severity of symptoms and virus variant. Interestingly, we found a strong decrease of the MAIT and CD8 Tr proportions coaligned with the disease severity. A decrease in the MAIT level has been previously associated with poor disease outcomes [16,38]. Reduction of the CD8 Tr cells probably reflects the accelerated transition of the less specialized CD8 Tr cells to the CD8 CT upon viral antigenic stimulation in the specific cytokine environment. Overall, changes in the lymphoid compartment affect CD8 and MAIT cell populations indicating strong displacement in the cellular immune responses in COVID-19 and identified deeper impairment of the cell composition in the cases with the Delta variant.

Based on our clustering and gene marker highlighting, we identified 5 cell states (Mon CD14, Mon CD16, Mon IFI30, Mon HLA, and pDC) associated with disease severity and coronavirus variant in the myeloid compartment (Figure 2C and Figure S3). The most impressive and significant changes were found among monocyte cell states (Mon IFI30) that are overrepresented in severe cases with the Delta variant (Figure 2A). The fraction of Mon IFI30 cells (CXCL8+, IFI30+, CD9+, and CD63+) is 150 folds lower (44% of monocytes for severe Delta cases, and only, 0.3% for Wuhan-like variant) in the PBMC of individuals with Wuhan-like variant compared to individuals with Delta. The molecular profile of Mon IFI30 cells is characterized by low expression of CD14, CD16, weak immunosuppressive (S100A8 and S100A9), and high inflammatory (LGALS, CTSB, and SPP1) gene signatures with an elevated level of cell trafficking and neutrophil activation agents (IFI30, CXCL8, and C15orf48). Furthermore, we observed a substantial decline of the CD14 and CD16 monocytes in samples with the Delta variant. Moreover, Mon CD14 cells are strongly overrepresented in severe samples with Wuhan-like virus but drop tenfold in Delta cases. We also identified depletion of the CD16 monocytes with a declining tendency toward an increase of the COVD-19 severity (Figure 2C). Overall, there are significant alterations both in myeloid and lymphoid cell subpopulations that reflect main immune changes and define specificities of the COVID-19 host–pathogen response. To further understand interdependencies in fractional changes between various cell states, we identified across sample negative correlation (Figure 2B) of the Mon IFI30 level with CD8 naive (r = −0.24) and MAIT (r = −0.33) cells pointing at a significant contribution to cellular immune systems response. Furthermore, we identified the lowest level of pDCs for Delta cases (Figure S4), indicating possible insufficiency of the specific immune response development leading to impairment and exacerbation of COVID-19 [39]. In conclusion, our analysis identified pronounced alterations in the myeloid cells that appear to be driven by the Delta variant of the SARS-CoV-2 and might be a valuable biomarker of prognosis and a potential therapeutic target.

### 3.3. Gene Expression Changes in the Severe COVID-19 Caused by Delta Variant

**Figure 3:**
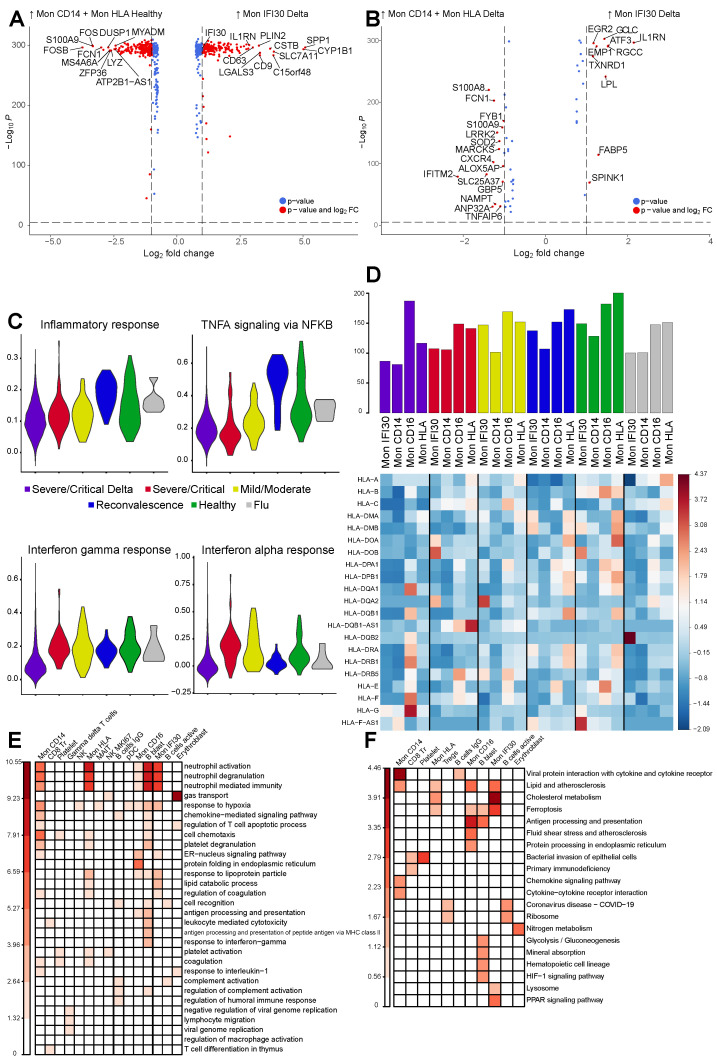
(**A**) Volcano plot showing gene expression changes between Mon IFI30 from severe Delta samples contrasted with Mon CD14 and Mon HLA from healthy groups because Mon IFI30 is enriched only in the severe Delta COVID-19. Only top significant markers with absolute logFC > 0.75 are highlighted. (**B**) Volcano plot showing gene expression aspects specific for Mon IFI30 contrasted with Mon CD14 and Mon HLA inside Delta samples and indicating gene activity heterogeneity in the myeloid PBMC group due to severe Delta COVID-19. Only top significant markers with absolute logFC > 0.75 are highlighted. (**C**) Violin plot with module scores for Inflammatory, TNF alpha, Interferon-gamma, and Interferon-alpha signatures for Mon IFI30 cells across study groups. (**D**) Heatmap with expression (z-score) of the HLA genes in monocytes across study groups. Bar plot with the summed expression of the HLA genes within a cell type in each cohorte. (**E**) Heatmap with enrichment of the Biological Processes and (**F**) KEGG pathways enriched in a set of upregulated (*p*.adj < 0.05, logFC > 1.5) genes obtained in a pairwise cell subtype comparison between severe Delta and Wuhan-like samples.

We then performed differential gene expression (DGE) analysis followed by GO enrichment calculation to reveal molecular factors and pathways specific for the severe form of COVID-19, depending on the virus variant. First, we focused on the whole monocyte compartment contrasting severe Delta samples with healthy ones. Because the Mon IFI30 population was enriched only in one group, we performed a comparison with combined Mon CD14 and Mon HLA. Our results indicate high expression of the IFI30, CD9, CD63, C15orf48, and LGALS3 in Mon IFI30 but monocytes of healthy individuals show high activity of FCN1, LYZ, MS4A6A, and DUSP1 (Figure 3A). Notably, overexpression of the CD9 and CD63 shows the involvement of extracellular vesicles in COVID-19 pathogenesis and can be associated with the faster virus spreading, mediating communication between infected and virus free cells [40,41]. In order to investigate what is specific for the Mon IFI30 compared to other PBMC monocytes in terms of gene activity, we contrasted them with Mon CD14 and Mon HLA within Delta cohort and identified elevated level of IL1RN, ATF3, LPL, EMP1, and EGR2 (Figure 3B).

Next, we focused on the expression differences and heterogeneity in the monocyte compartment between the severe cohortes. We contrasted Delta Mon IFI30 with Mon CD14 and Mon HLA from severe Wuhan-like samples (Figure S5). We identified that the most differential genes in Mon IFI30 include IL1RN, ATF3, LPL, EGR2, and CXCR4, S100A8, S100A9, IFITM2, and FCN1 are more active in the Mon CD14 and Mon HLA from Wuhan-like group. Many of these genes are associated with proinflammatory properties and might have an important role in immune response for COVID-19. Furthermore, single nucleotide polymorphisms located in IL1RN are associated with COVID-19 severity [42,43], and S100A8/A9 appear to be a potential progression biomarker. We found S100A8/A9 are overexpressed in the Wuhan-like but not Delta samples which highlights the importance of the additional investigations covering other SARS-CoV-2 variants.

To better understand expression programs in each cell type depending on the virus variant, we performed an analysis aimed to clarify the role of the gene subsets with respect to certain functions or processes. Particularly, previous studies identified a strong association of the COVID-19 severity with IFN type I and TNF alpha responses [10,44,45]. Interestingly, we found a highest depletion of the IFN signatures for Delta samples but not for the severe Wuhan cases with the highest level of IFN alpha signatures (Figure 3C). However, TNF alpha gene signatures are generally higher in reconvalescent and healthy groups and are lowest in severe Wuhan cases (Figure 3C). Previously, an increase of the IFN alpha signatures of severe/critical patients was shown compared to the healthy group [46], but we identified opposite for the patients with Delta variant. We also identified that enrichment of IFN and TNF alpha gene signatures for Mon HLA in the Delta group are inline with Mon IFI30 (Figure S6). Furthermore, pDCs are the producers of IFN and significantly depleted cell types in Delta samples (Figure S4). Thus, their reduced level positively correlates with a decrease in the IFN signatures.

Previous studies described changes in HLA expression and linked decrease of the abundance with severity of the COVID-19 [47]. Here, we investigated expression of the HLA gene panel (Figure 3D) for monocytes from various cohorts. We noted reduction of the HLA expression for Mon IFI 30, Mon CD14, and Mon HLA in severe groups with Delta lineage when compared with influenza, healthy, or other COVID-19 samples. Surprisingly, Mon CD16 shows an opposite pattern with the highest HLA abundance. Altogether, these results are inline with previous studies where authors observed less efficient immune response due to diminished antigen presentation potential of monocytes that are also gaining inflammatory phenotype [48].

We performed comparison of gene expression between Delta and severe Wuhan-like samples followed by enrichment analysis to identify molecular processes (Figure 3E) and pathways (Figure 3F) associated with the sets of differentially active genes. We focused on expression brought by virus variants to identify hallmark pathways in each cell type. Contrasting both severe groups of patients with healthy samples, we identified activation of neutrophil chemotaxis, hypoxic response, and immune-associated processes, indicating development of the antiviral activity (Figure S7). Interestingly, direct gene expression comparison between Delta and Wuhan-like groups result in high activity of the pathways involved in neutrophil degranulation (Figure 3E). Our results show that all subtypes of monocytes interplay with neutrophils and have elevated levels of genes associated with leukocyte-mediated immune response and degranulation. Moreover, activated in the monocytes genes showed high involvement in chemotaxis and coagulation (Figure 3E). We want to stress a point that activation of neutrophils and degranulation processes is a commonly activated pathway in severe COVID-19 [11]. Thus, Delta samples show extra involvement of neutrophils in immune responses mediated by monocytes. Altogether, this leads to a detrimental loop involving the release of cytokines and recruitment of cytotoxic damaging cells to tissues [11]. Moreover, we also observed activation of coagulation processes and complement systems according to pathway analysis for upregulated genes in Mon CD14, Mon HLA, platelets (Figure 3E). Elevated activity of the PPAR signalling pathways in Mon IFI30 (Figure 3F) cells can be associated with formation and release of extracellular vesicles [49]. Taken together, we conducted an integrative gene expression analysis and can conclude that all subtypes of monocytes contribute to a higher rate and severity of the COVID-19 exacerbations for the Delta variant.

### 3.4. Gene Regulatory Network Governs Specific Monocyte States in Delta COVID-19

**Figure 4:**
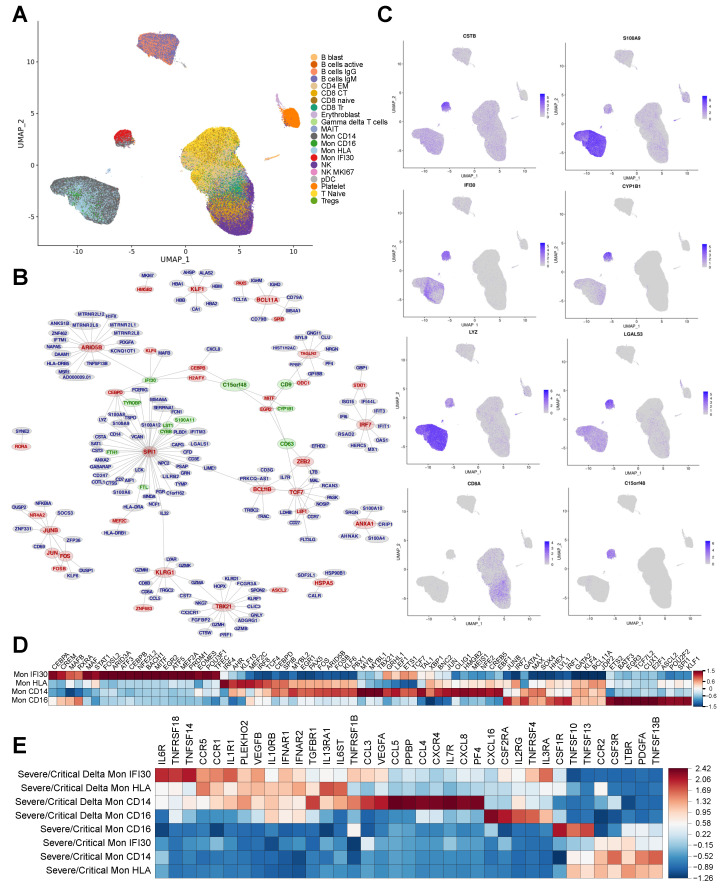
(**A**) UMAP with colored cell annotation as obtained based on the CCA integration. Cell placing on the plot reflects similarities and colocalization of the cell types. (**B**) Fragment of the gene regulatory network reconstructed based on single–cell gene expression obtained for the whole dataset. Top 200 gene regulators (TFs) with their the most confident targets are plotted. Red color highlights important regulators that were recovered based on the SCENIC gene regulatory network reconstruction. Green labels show markers of the Mon IFI30 cell subtype. Network visualized using Fruchterman–Reingold layout algorithm. (**C**) Expression of the marker genes as projected on the SCENIC–based UMAP indicates activity of the core marker genes and colocalization of the cell subtypes. Mon IFI30 marker genes (LGALS3, CSTB, and C15orf48) are highly expressed in the population that was previously identified using CCA integration and clustering. (**D**) Heatmap with average gene expression for transcription factors that are differentially expressed (logFC > 0.75) in each cell type with respect to other monocytes. (**E**) Heatmap with average cytokine expression in monocytes for Delta and Wuhan-like samples.

Changes in gene expression are caused by complex regulatory mechanisms that maintain the functionality and adaptivity of cells concerning environmental conditions [50]. The major core of this regulatory landscape are transcription factors usually working in a DNA-binding manner and control the expression of target genes [51,52]. We performed reconstruction of the transcriptional Gene Regulatory Network (GRN) applying SCENIC [25]. First, we verified that Mon IFI30 cells are indeed mainly present in severe/critical patients with the Delta variant (Figure S8A,B). Performing cell clustering on top of the reconstructed GRN, we identified that Delta-specific monocytes (Mon IFI30) indeed group to a separate cluster (Figure 4A). Moreover, on top of the SCENIC based UMAP representation, we colored cell types identified based on Seurat CCA integration [22] and observed a clear visual cell separation of Mon IFI30 based on previous annotation (Figure S9). Thus, clustering based on the predicted regulons enables the identification of cell states with clear separation of the Delta-variant-associated monocytes to distinct clusters only for one cohort (Figure S9). Overall, these results shed additional light on the regulatory role of transcription factors involved in generating a particular cell state and lead to a better understanding of complex gene expression program perturbations in COVID-19 caused by the Delta variant.

To verify GRN-based clustering approach, we found that known FOXP3 TF governing the development of Tregs was also captured, even considering the low abundance of these cell types in PBMC (Figure S10). GRN reconstruction yields that CEBPB, MITF, and SPI1 are core regulators of the Mon IFI30 subtype (Figure 4B). In order to gain additional confirmations, we studied the expression of the regulatory TFs in subtypes of monocytes. Indeed, each monocyte state (Mon IFI30, Mon CD16, Mon CD14) is characterized by the activity of a specific set of TFs that were predicted to regulate cell state marker genes (Figure 4C,D). Such significant changes in the transcriptional regulatory landscape indicate profound phenotypic differences. Mainly, ATF3, MITF, CEBPB, NFE2L2, BACH1, STAT1, and MAF are highly expressed in the Mon IFI30 (Figure 4D) and are known to be involved in the development of immune reactions—interferon type I response and upregulation of proinflammatory cytokines [53,54,55,56]. Moreover, we also identified that these genes are differentially expressed between monocyte subtypes. Mon CD14 and Mon HLA show similar gene expressions for most TFs. However, Mon HLA demonstrates higher levels of the IRF4 and IRF8 compared to the Mon CD14 with upregulated MYB and LEF1 (Figure 4D). For Mon IFI30, we also found MAFB and MAF as highly expressed and previous studies proposed their role for control of macrophage checkpoints in COVID-19 severity and as a marker for progression [18]. MITF was also discovered as a potential indicator of the COVID-19 severity [57]. Altogether, discovery of transcriptional regulators highlights a possibility to be used as a potential predictive biomarker or pathway for targeted treatments [58].

We revealed strong differences in the level of produced cytokines, clearly clustering monocytes inside Delta from Wuhan-like cases and indicating differences in cytokine expression between monocyte subtypes within the sample group (Figure 4E). Mon HLA and Mon CD14 inside Delta samples differ in expression for the cytokine panel (CCL3, VEGFA, CCL5, CCL4, CXCR4, IL7R, CXCL8, and PF4) that have been found associated with COVID-19 severity and mortality [59]. Altogether, this analysis yields that the severity of the Delta variant can not be explained only by the abundant subtype of monocytes (Mon IFI30), because cells coexist in complex interactions and regulations that ultimately affect gene expression and phenotype of other cells.

### 3.5. Ligand-Receptor Cell–Cell Communications in Severe Delta COVID-19

The development of severe/critical COVID-19 strictly depends on the interactions between components of the immune system via direct cell–cell contacts and ligand–receptor interactions. Here, we applied CellChat [26] to single-cell gene expression data and predicted cell–cell communications (CCC) and differential ligand–receptor interactions interplay between severe/critical COVID-19 cases depending on the virus variant.

We performed analysis of the differential ligand–receptor interactions and identified that both severe Delta and Wuhan-like cases demonstrate significant alterations of the cell–cell communication pattern. We contrasted the Delta and healthy groups and predicted a high number of gained CCCs established mainly between myeloid and B-cells, Tregs, and T naive groups (Figure S11A). However, CD8 Naive cells show loss of CCCs with NK groups but more mature CD8 CT and CD8 Tr gain new interactions with pDC. Differentially gained interactions for severe Wuhan cases occur mostly between Mon CD14 and B cells (B cells IgG, B cells IgM, and B cells active), pDC, and Mon CD16 (Figure S11A).

**Figure 5:**
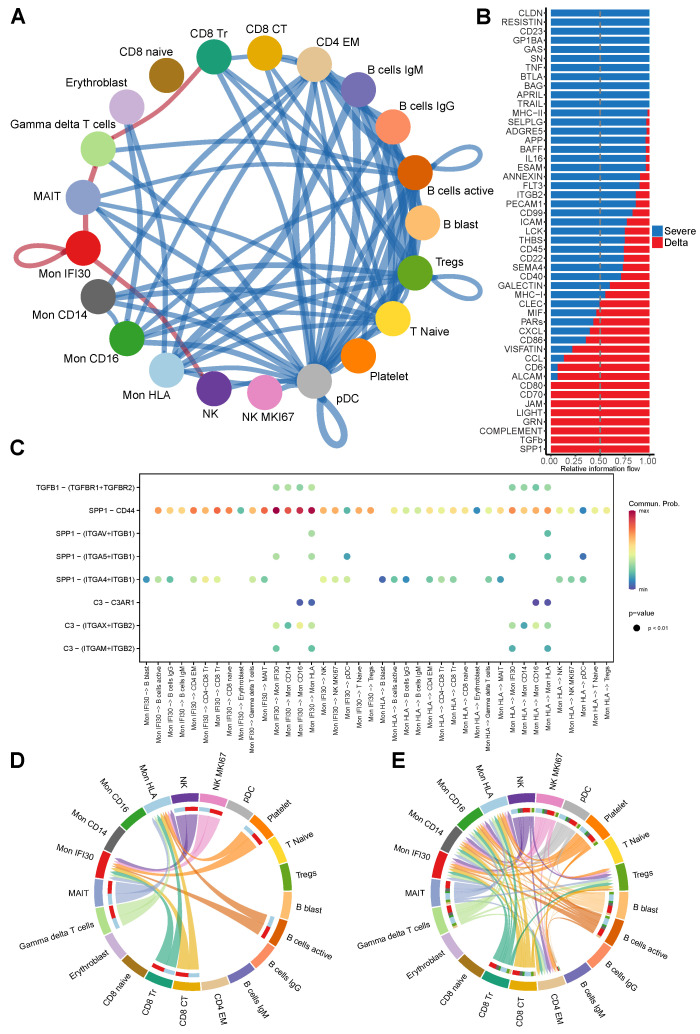
(**A**) Circle plot showing predicted differential cell–cell communications between severe Delta and Wuhan-like groups (red for gained, and blue for lost in Delta cases). We identified several newly gained communications for the Delta cohort established by Mon IFI30 with MAIT and NK cells and by gdT-cells with MAIT and CD8 Tr cells. (**B**) Bar plot showing the intensity of context-specific signaling pathways measured as relative information flow as implemented in CellChat. TGF beta and SPP1 are predicted as main signaling molecules involved in cell–cell communication for severe Delta COVID-19 aside from Resistin, CD23, and Caledon. (**C**) Dot plot showing cell pairs (x-axis) and predicted interacting ligand–receptors (y-axis) for SPP1, TGF beta, and complement pathways. Color on the dot plot shows the strength of a given ligand–receptor interaction quantitatively represented by a probability value obtained with CellChat and calculated based on the law of mass action relying on the average expression of the ligand in a cell group and receptor by another cell group. (**D**) Chord diagram showing interacting cell types in severe samples via CCL-CXCL axis for severe Wuhan-like samples. (**E**) Chord diagram showing interacting cell types via CCL-CXCL axis for severe Delta samples.

We also directly contrasted severe Delta and Wuhan-like cases to identify the most diverging ligand–receptor interactions. Notably, the most intensive differentially gained interactions for Delta samples involve cell–cell communication of Mon IFI30 with NK and MAIT cells and also between gdT-cells with MAIT and CD8 Tr cells (Figure 5A). Besides newly gained interaction, we also predicted the pronounced loss of the interactions between myeloid and lymphoid cells. Mon CD14 experiences differential loss of interactions with CD4 EM, Tregs, and T naive cells. Mon HLA shows depletion of interactions with B cells IgM, B cells IgG, and Tregs. Notably, we predicted that the pDC subset lost ligand–receptor interactions with most of the PBMC cell types, which can be explained by significant decrease of the pDC cell fraction for severe COVID-19 patients with the Delta variant.

We also performed analysis of main signaling pathways that govern cell–cell communications in both severe groups depending on the virus variant. In the case of Delta lineage, we found SPP1 and TGF beta as core involved pathways (Figure S11). However, for Wuhan-like cases, Claudin and Resitin are main cell communication molecules. Furthermore, both severe groups demonstrate strong involvement of protease-activated receptors (PARs), which are considered as drug target candidates for COVID-19 treatment [60]. We performed direct comparison of severe groups with each other to identify differential signaling pathways governing cell–cell communications in Delta contrasted to Wuhan-like samples. In particular, ligand–receptor interactions between immune cells in the case of the Delta variant are strongly driven by SPP1, TGF-β, and complement proteins (Figure 5B). However, in the severe group with the Wuhan-like virus, the most intensive differential communication signal comes from Resistin, Caledon, CD23, and TNF (Figure 5B).

We investigated further ligand–receptor interactions established via main differential pathways (SPP1, TGF-β, complement) for immune cells in the Delta samples. We observed a broad spectrum of communication between Mon IFI30 cells, lymphocytes, and monocytes. However, high fidelity communications via TGF-β and C3 complement pathways occur only within monocytes (Figure 5C). Next, we investigated interactions established via SPP1 that strongly overactivated in Delta but not Wuhan-like samples. The most abundant SPP1 interactions are mediated via the SPP1-CD44 pair, where the highest communication probability has been identified between monocyte subtypes, including autocrine loops (Figure 5C).

We further looked at perturbations of the CCL and CXCL pathways and found that patterns of interactions for Delta samples partially overlap with Wuhan-like severe cases (Figure 5D,E). In general, compared to Wuhan-like, Delta cases have much more pronounced interactions involving monocyte subtypes. We dissected CCL interactions further and found that PF4-CXCR3, CCL5-CCR1, and PF4V1-CXCR3 are common ligand–receptor pairs both for severe Wuhan-like and Delta COVID-19 cases (Figure S12A,B). However, in the Delta samples, interactions via mentioned ligand–receptors involve more cell type pairs.

In addition, we identified that CCL and CXCL pathways involve interactions via the CCL5-CCR4 pair, and our predictions indicate implication only of the lymphoid but not myeloid cell types (Figure S12A). Previous studies showed an increase of inflammatory CXCR4+ T cells in the lungs of severe COVID-19 patients, supporting a hypothesis that lung-homing T cells contribute to immunopathology, and nonsuppressive T cells restrict pathogenesis and are involved in recovery from severe COVID-19 [61].

Last, we trained a binary classifier (AuROC = 0.95) to distinguish monocytes in Delta and Wuhan-like samples based on the ligand and receptor expressions (see Section 2). High classification accuracy coupled with SHAP analysis [62] yields top important for classification molecules (Figure S13A). Moreover, we identified that every type of monocytes can be clustered based on the disease-causing virus variant relying on the expression profile of the ligand–receptor pairs (Figure S13B). We revealed SPP1 and C3 ligands as the most associated with monocytes in Delta cases, and SIGLEC1 and CD74 are strong predictors of the monocytes from severe Wuhan-like samples. Interestingly, TGFB1 achieves the highest expression for Mon IFI30 in Delta samples (Figure S13C), and all myeloid PBMC cells in the Delta cohort experience elevation of TGFB1 and TGFBI and receptors TGFBR1 and TGFBR2 compared to Wuhan-like group (Figure S13C). Mon CD16 highly expresses the receptors that point at Delta specific immune dysregulation established by Mon IFI30 via TGF beta. Overall, identified expression patterns of the ligand–receptor pairs uncover the main molecular pathways involved in developing virus lineage-specific immune response in COVID-19. Our results point to a significant impact of the Mon IFI30 to CCC, highlighting significant involvement of SPP1 and TGF-β.

## 4. Discussion

Most cases of SARS-CoV-2 have an asymptomatic or mild form. Up to 15% cases develop severe pneumonia and about 5% may have more severe conditions with multiple organ failure [63].

Moreover, circulating SARS-CoV-2 differs in virus transmission, impact on the immune response, and risk of reinfection. Cytokine storms that develop during COVID-19 lead to a robust immunological shift resulting in a switch to a proinflammatory program of myeloid cells both in blood and lung tissue [64,65], compositional dysregulation in the lymphoid compartment. Investigation of immune responses in COVID-19 patients is a primary goal to achieve the effectiveness of treatments, define disease prognosis markers, and understand the heterogeneity of disease severities. We utilized computational methods to combine clinical observations and single-cell transcriptomes and develop a comprehensive, integrated view of COVID-19 immune response depending on the severity of symptoms and SARS-CoV-2 virus variant.

Here, we generated and analyzed a large single-cell transcriptomic atlas of blood mononuclear cells from 40 newly sequenced individuals combined with 36 published samples. In order to gain insight into immune response differences that SARS-CoV-2 variants bring to the immune response, we performed an investigation of PBMC samples with Wuhan-like or Delta (B.1.617) virus variants that we also compared with severe Flu yielding different immunological mechanisms. We applied scRNA-seq profiling to investigate the molecular programs associated with COVID-19 severity and the SARS-CoV-2 variant. Our current focus on the transcriptional description of gene expression identified subtypes of monocytes (Mon IFI30) that were strongly enriched (150 fold) in the samples with the Delta variant. We observed impressive immunological differences specific to severe COVID-19 caused by the Delta variant. We revealed that Mon IFI30 cells have a high expression of chemokines (CXCR4, CXCL8, and CCL3), mediating chemotaxis and cell migration, and proinflammatory (CSTB, LGALS3, SPP1) factors. Previous studies showed high overlap of the upregulated inflammatory signatures in the PBMCs from COVID-19 and HIV patients [66]. Despite varied gene expression responses depending on the infection agent, various subset of immune cells might share signatures common with other conditions also bringing their own features. Other investigations identified that developed in COVID-19 sepsis cytokine storm was less pronounced but with higher immunoglobulin and complement protein levels compared to bacterial sepsis patients [67]. The Mon IFI30 subtype is characterized by a specific gene expression profile clearly indicating that severe Delta variant COVID-19 leads to specific immune shifts. A significant impact can bring possible mechanisms of the fast virus spreading, involving extracellular vesicles activated in Mon IFI30.

We also confirmed that Mon IFI30 cells demonstrate a particular TFs expression profile. We identified that CEBPB, MITF, and SPI1 are involved in the regulation of marker genes responsible for chemotaxis and neutrophil activation (CXCL8, IFI30). However, Mon HLA cells indeed show expression similar to Mon CD14 with elevation of the IRF4 and IRF8, indicating more robust interferon signaling. Furthermore, Mon IFI30 cells have elevated levels for CD9 and CD63 complemented with PPAR signaling that altogether might be involved in faster virus spreading via receptors (CD9 and ACE2), making cells susceptible for virus entrance [41].

We also consider certain limitations of our study coming from investigating the severe Delta cases only, which are caused by our accessibility to human samples.

We refer to single-cell samples [7] of patients with severe Wuhan-like COVID-19, but the virus genotyping has not been performed and strain is suggested based on the information about GEO submission date (September 2020), which is much earlier than the Delta variant was first detected (December 2020). Besides gene expression investigation, a more profound validation is required, which can be brought to the field by open chromatin profiling using scATAC-seq data. Moreover, broader investigations of the identified subtype of monocytes require additional study of extracellular vesicles involvement in pathogenesis of severe COVID-19 caused by other variants of the virus.

## Data Availability

Access to raw and processed datasets will be provided for research community before publication.

## Supplementary Materials

The following supporting information can be downloaded at: https://www.mdpi.com/article/10.3390/cells11192950/s1, Figure S1: UMAP coloured by predicted celltype using Symphony computational approach, Figure S2: Comparison of the Seurat CCA and Harmony data integration approaches, Figure S3: Feature plots, Figure S4: Changes of the immune cell fractions in PBMC, Figure S5: Scatter plot, Figure S6: Module scores for gene signatures, Figure S7: Gene ontology terms, Figure S8: SCENIC UMAP splitted by severity, Figure S9: SCENIC UMAP plots for individually processed cohortes, Figure S10: Entire gene regulatory network, Figure S11: Cellchat differential interactions, Figure S12: Dotplot for communication molecules of the CCL-CXCL pathway, Figure S13: Ligand and receptors contrasting severe Delta and Wuhan-like cases based on the gene expression in monocytes, Table S1: Description of the in-house and public datasets used in the study.
