# Supplementary material for: Single-Cell Gene Expression Analysis Revealed Immune Cell Signatures of Delta COVID-19
Source: Cells. 2022 Sep 21;11(19):2950. doi: 10.3390/cells11192950 (PMC9563974; doi:10.3390/cells11192950)
Supplement: Supplementary file 1 [file cells-11-02950-s001.zip › supfiles/S5 copy.pdf]

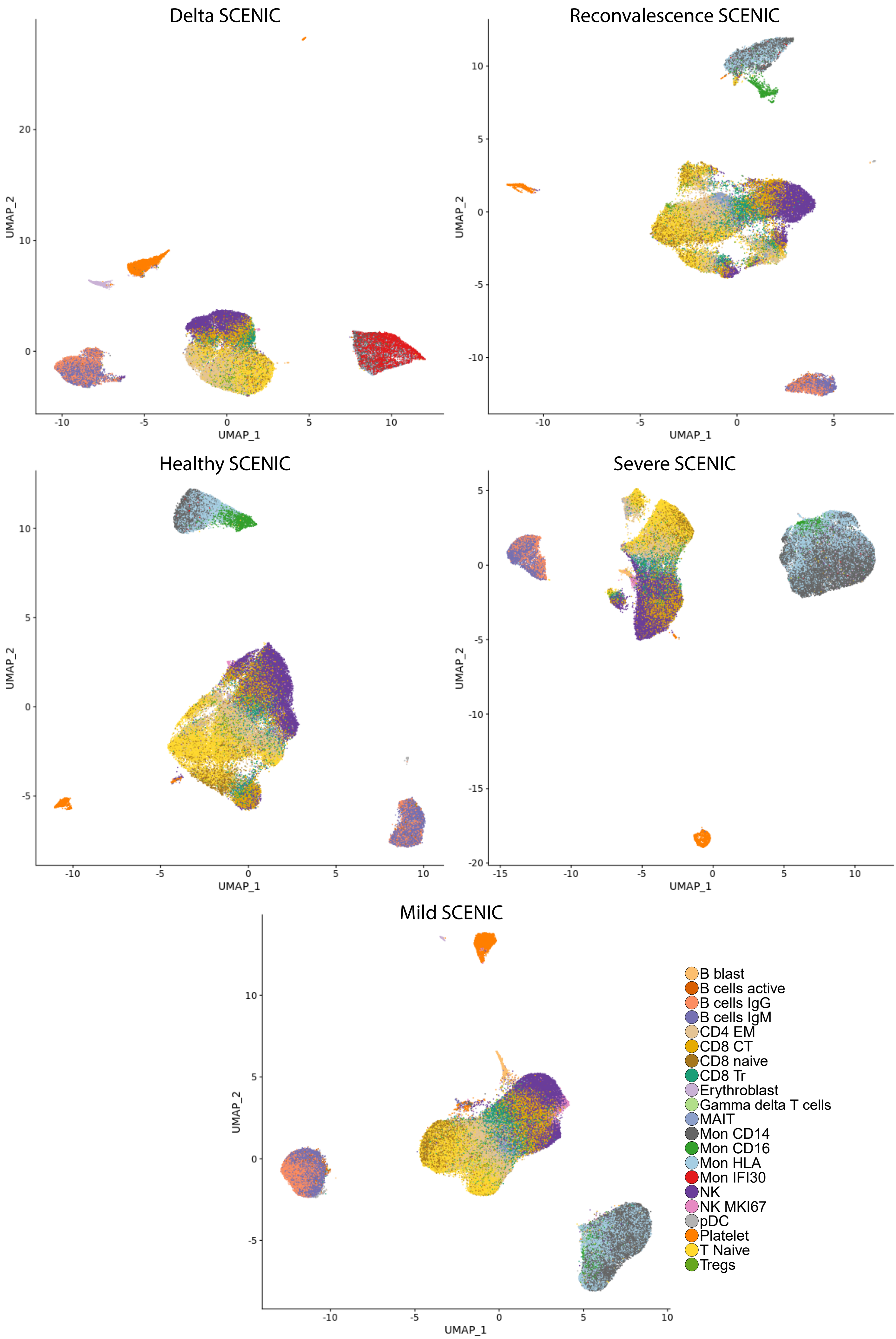

Delta SCENIC
Reconvalescence SCENIC
Healthy SCENIC
Severe SCENIC
Mild SCENIC
UMAP_1
UMAP_2
B blast
B cells active
B cells IgG
B cells IgM
CD4 EM
CD8 CT
CD8 naive
CD8 Tr
Erythroblast
Gamma delta T cells
MAIT
Mon CD14
Mon CD16
Mon HLA
Mon IFI30
NK
NK MKI67
pDC
Platelet
T Naive
Tregs
