# Supplementary material for: Single-Cell Gene Expression Analysis Revealed Immune Cell Signatures of Delta COVID-19
Source: Cells. 2022 Sep 21;11(19):2950. doi: 10.3390/cells11192950 (PMC9563974; doi:10.3390/cells11192950)
Supplement: Supplementary file 1 [file cells-11-02950-s001.zip › Shaymardanov_paper_supp_final.pdf]

Supplementary Materials: Single-cell gene expression analysis revealed immune cell signatures of Delta COVID-19

Abusaid M. Shaymardanov[1,†], Olga A. Antonova[1,†], Anastasia D. Sokol[1], Kseniia A. Deinichenko[1], Polina G. Kazakova[1], Mikhail M. Milovanov[1], Alexander V. Zakubansky[1], Alexandra I. Akinshina[1], Anastasia V. Tsypkina[1], Svetlana V. Romanova[1], Vladimir E. Muhin[1], Sergey I. Mitrofanov[1], Vladimir S. Yudin[1], Sergey M. Yudin[1], Antonida V. Makhotenko[1], Anton A. Keskinov[1], Sergey A. Kraevoy[1], Ekaterina A. Snigir[1], Dmitry V. Svetlichnyy[1,‡*], Veronika I. Skvortsova[2]

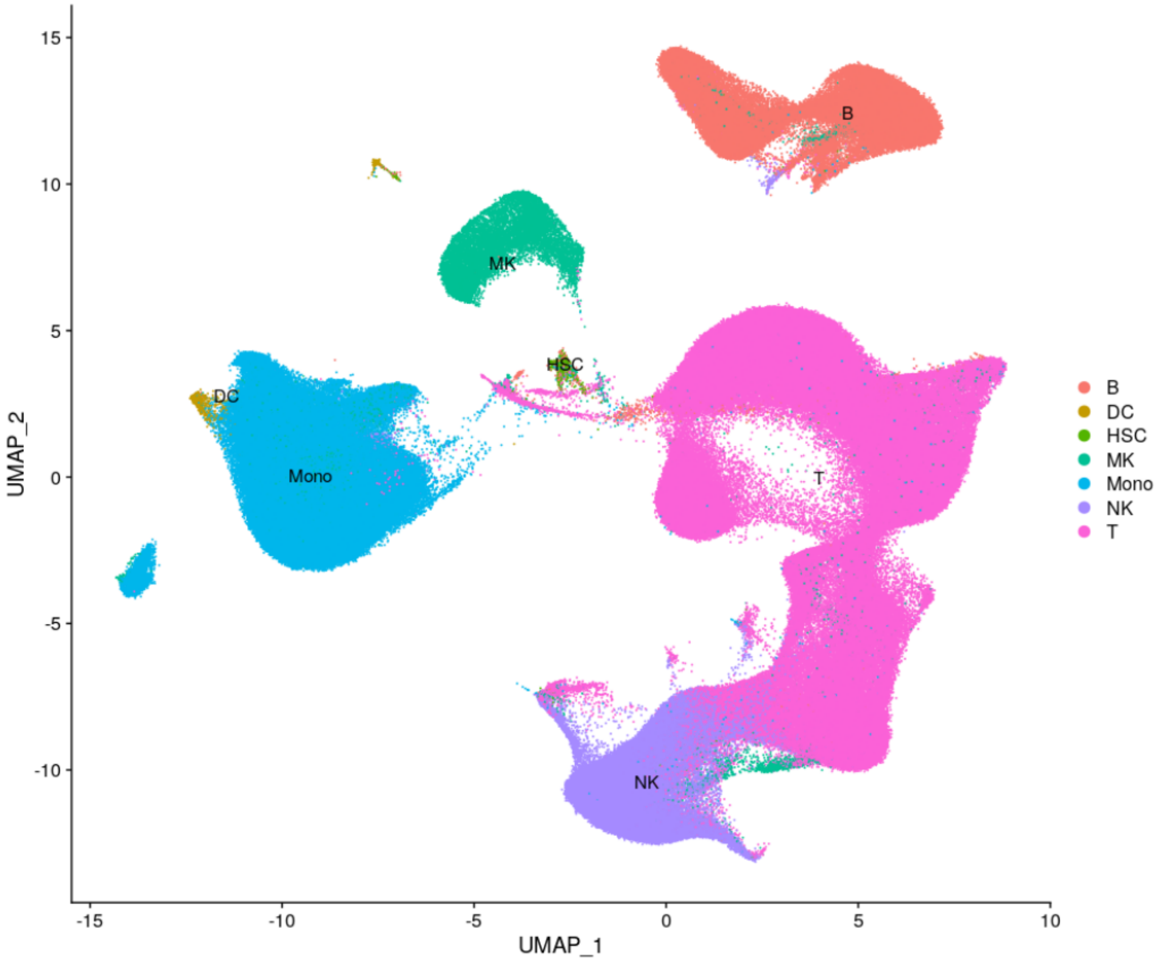

Figure S1. UMAP coloured by predicted celltype using Symphony computational approach. The UMAP plot demonstrates concordance with manual annotation. We applied label transfer from a dataset based on annotated 10X PBMC scRNA-seq (REF). Clear separation of the clusters show colocalisation of the main cell types (B-cells, Monocytes, T-cells, NK, megakaryocytes, DCs)

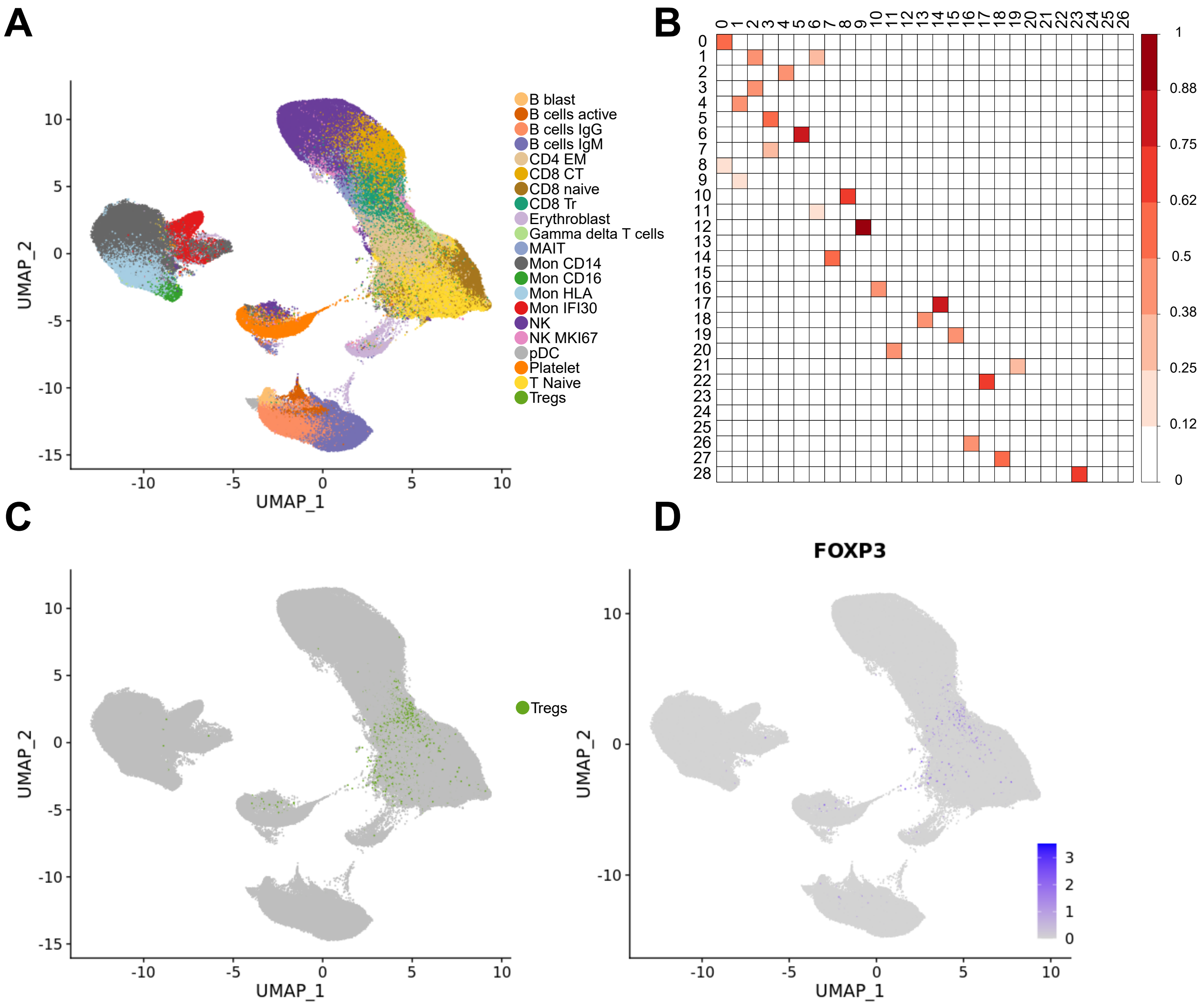

Figure S2. Comparison of the Seurat CCA and Harmony data integration approaches. (**A**) Harmony UMAP plot colored by cell subtypes demonstrates concordance with CCA integration and topological colocalisation of the annotated cell types. (**B**) Heatmap with Jaccard index identifies high similarity between major clusters obtained with Seurat CCA and Harmony. (**C**) Treg cell demonstrate disseminated localisation within T-cells compartment. (**D**) FOXP3 expression is highlighted on the UMAP.

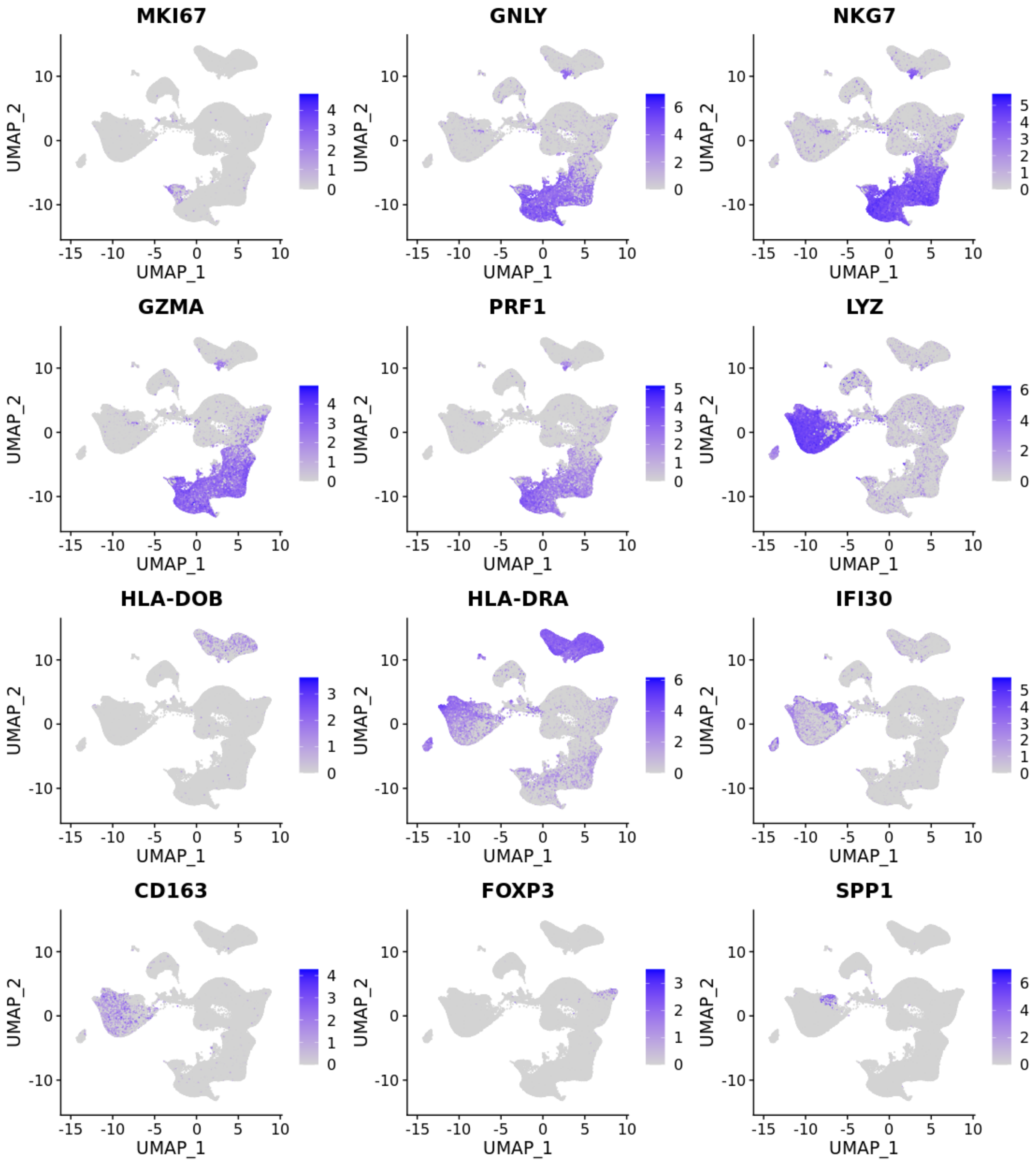

Figure S3. Feature plots. Feature plot with gene expression projected on the CCA UMAP plot.

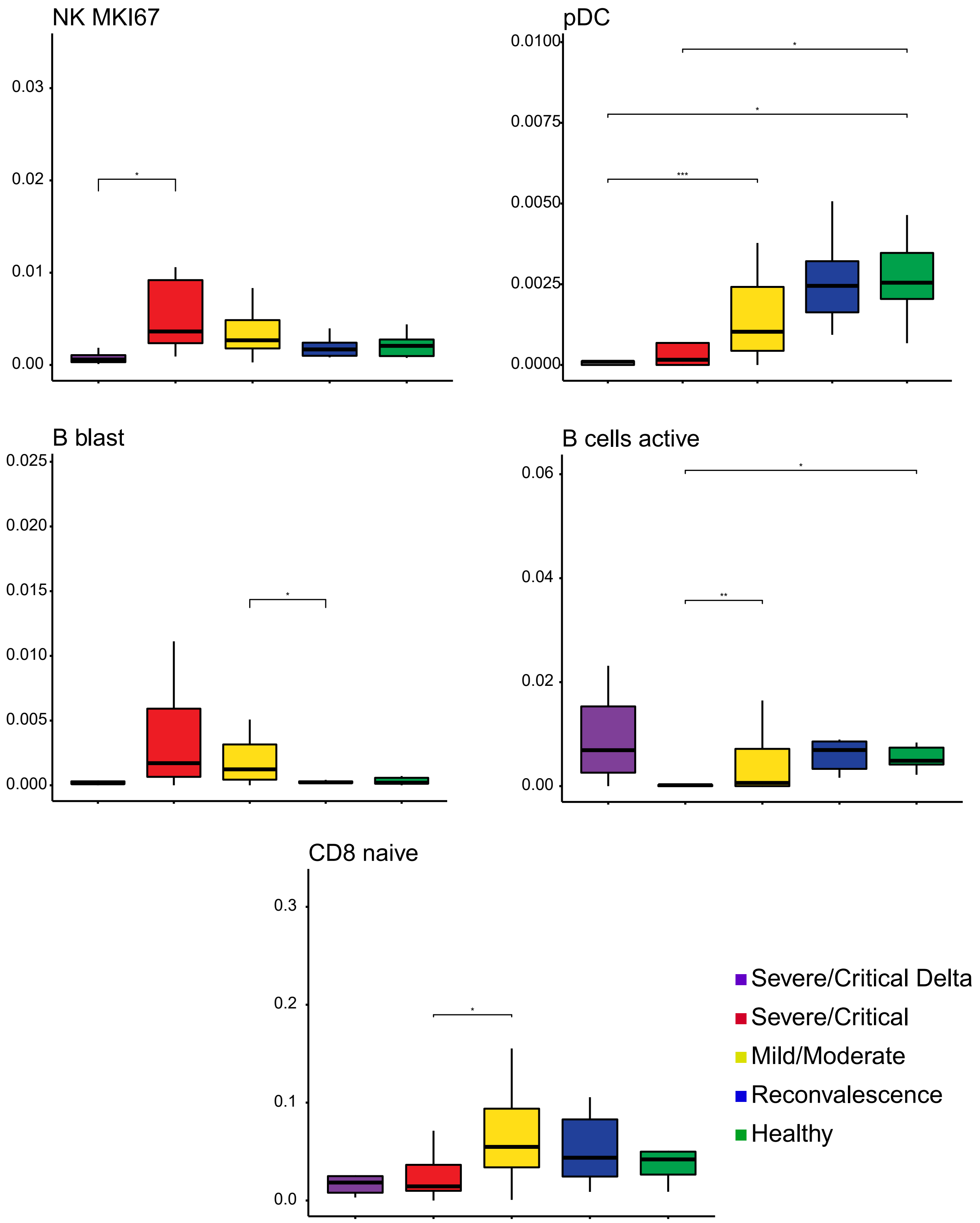

Figure S4. Changes of the immune cell fractions in PBMC. Boxplots with changes of the cell fractions for immune subtypes. P-value was calculated using Wilcoxon rank-sum test.

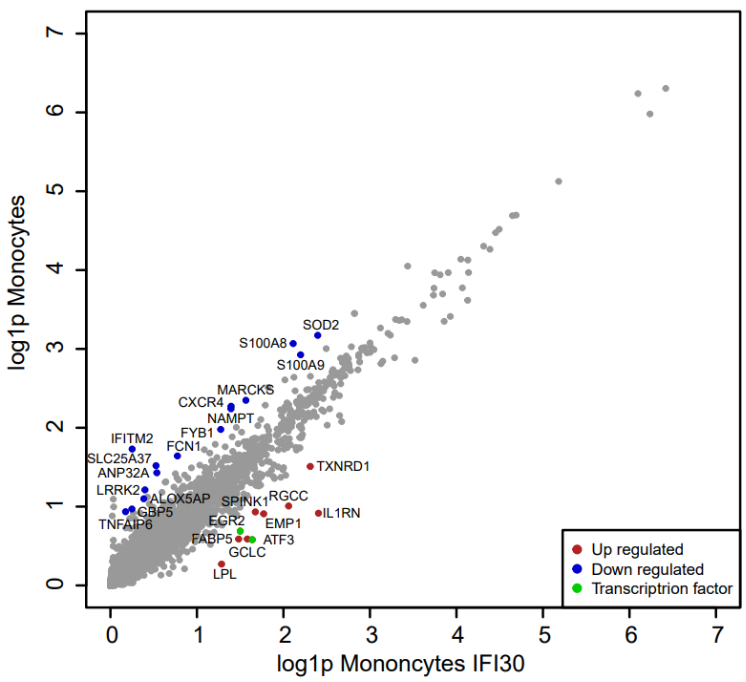

Figure S5. Scatter plot showing gene expression changes between Mon IFI30 and other monocytes (Mon CD14, Mon HLA) across all study cohortes. Significant up/downregulated genes are highlighted (logFC>2, padj<0.05).

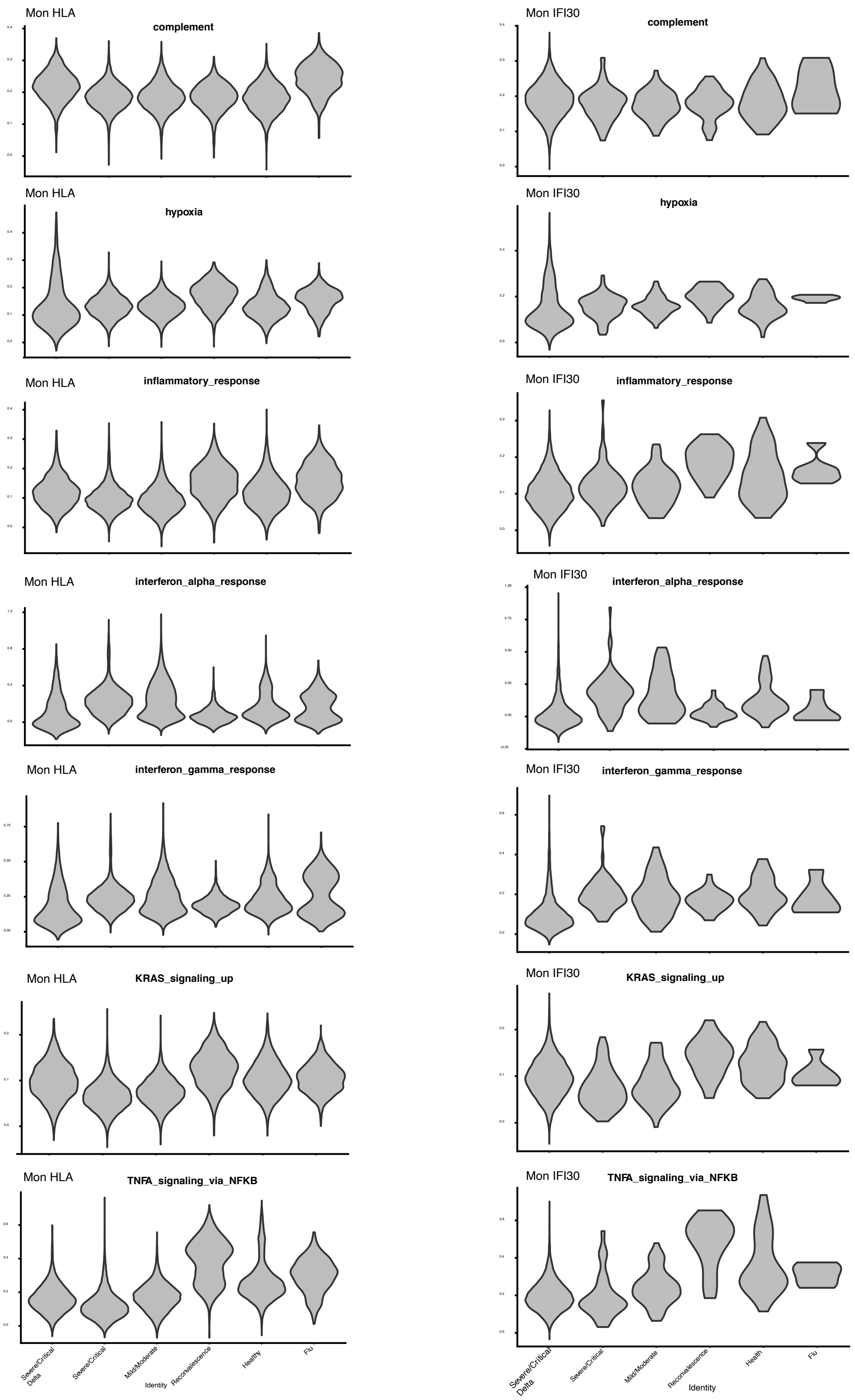

Figure S6. Module scores for gene signatures. Violin plots with the gene module scores distribution in Mon IFI30 and Mon HLA across 6 study groups.

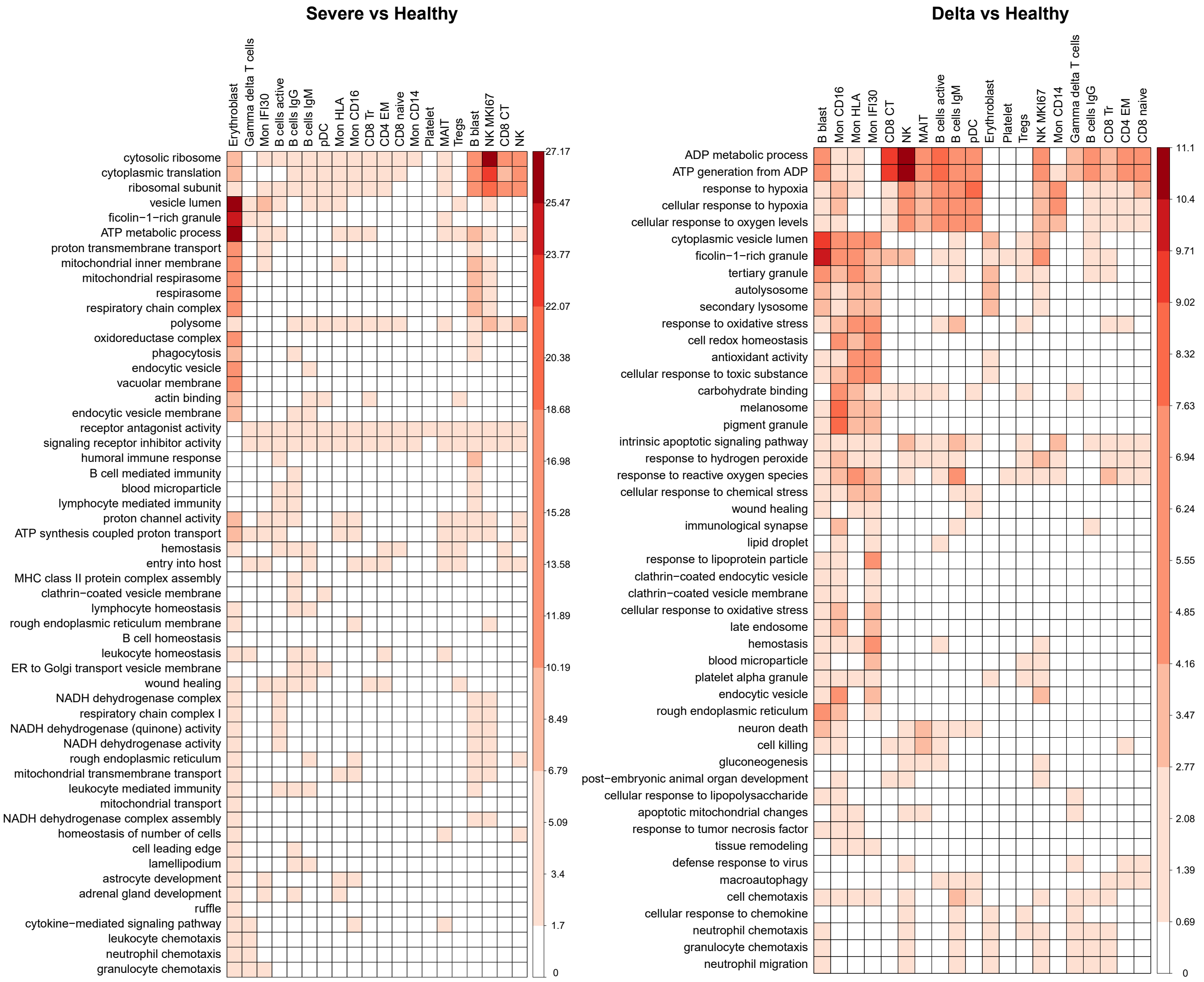

Figure S7. Gene ontology terms. Gene ontology overrepresentation calculated based on up-regulated expressed genes (logFC > 1, padj<0.05) obtained in a pairwise comparison of the common cell subtypes between severe (Delta or Wuhan-like variants) and healthy individuals.

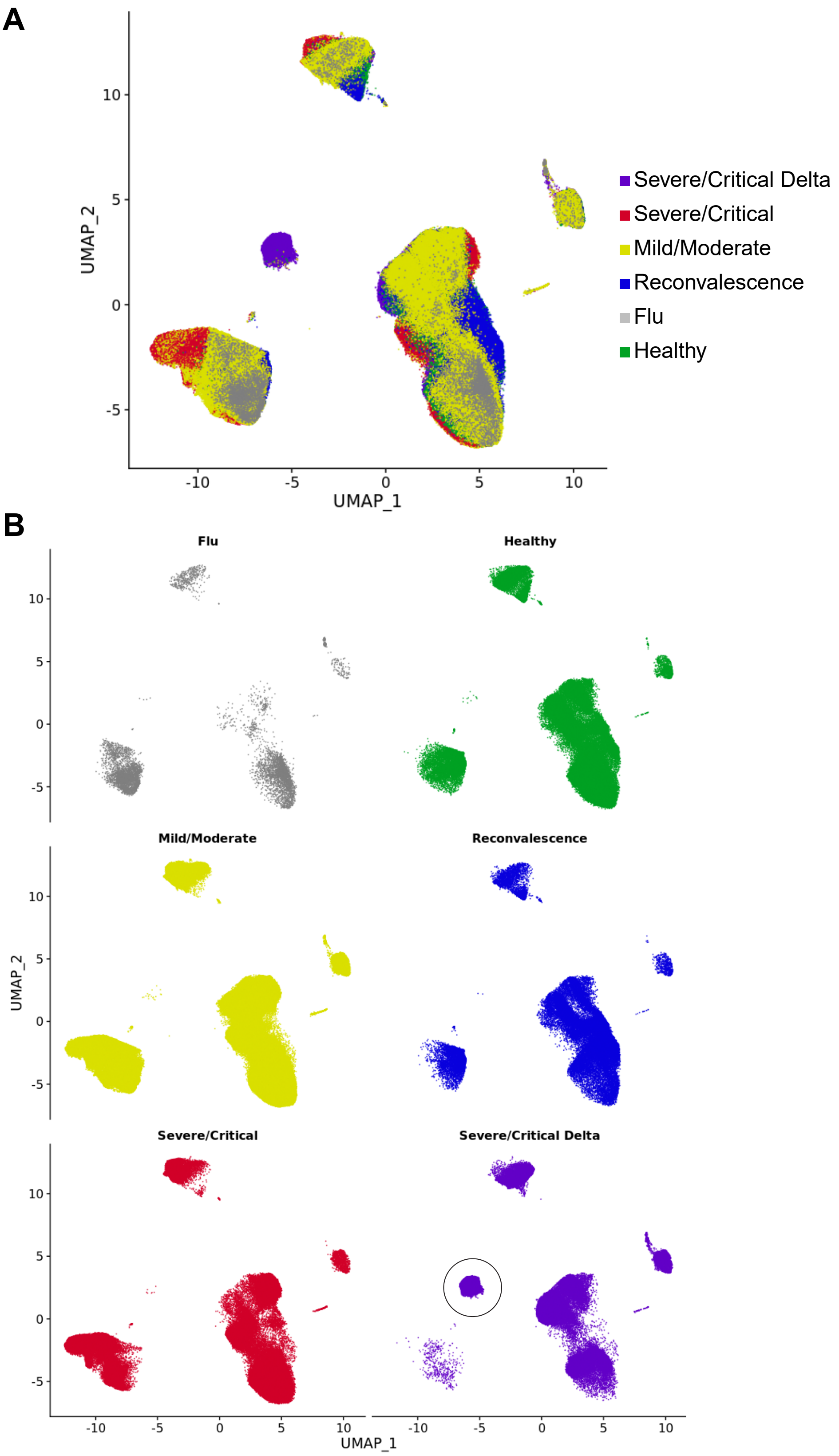

Figure S8. SCENIC UMAP splitted by severity. (**A**) UMAP plot based on combined dataset colored by cohorte types. (**B**) UMAP plot based on SCENIC combined dataset splitted by cohortes. Cell subtype enriched only in the severe Delta samples highlighted with circle.

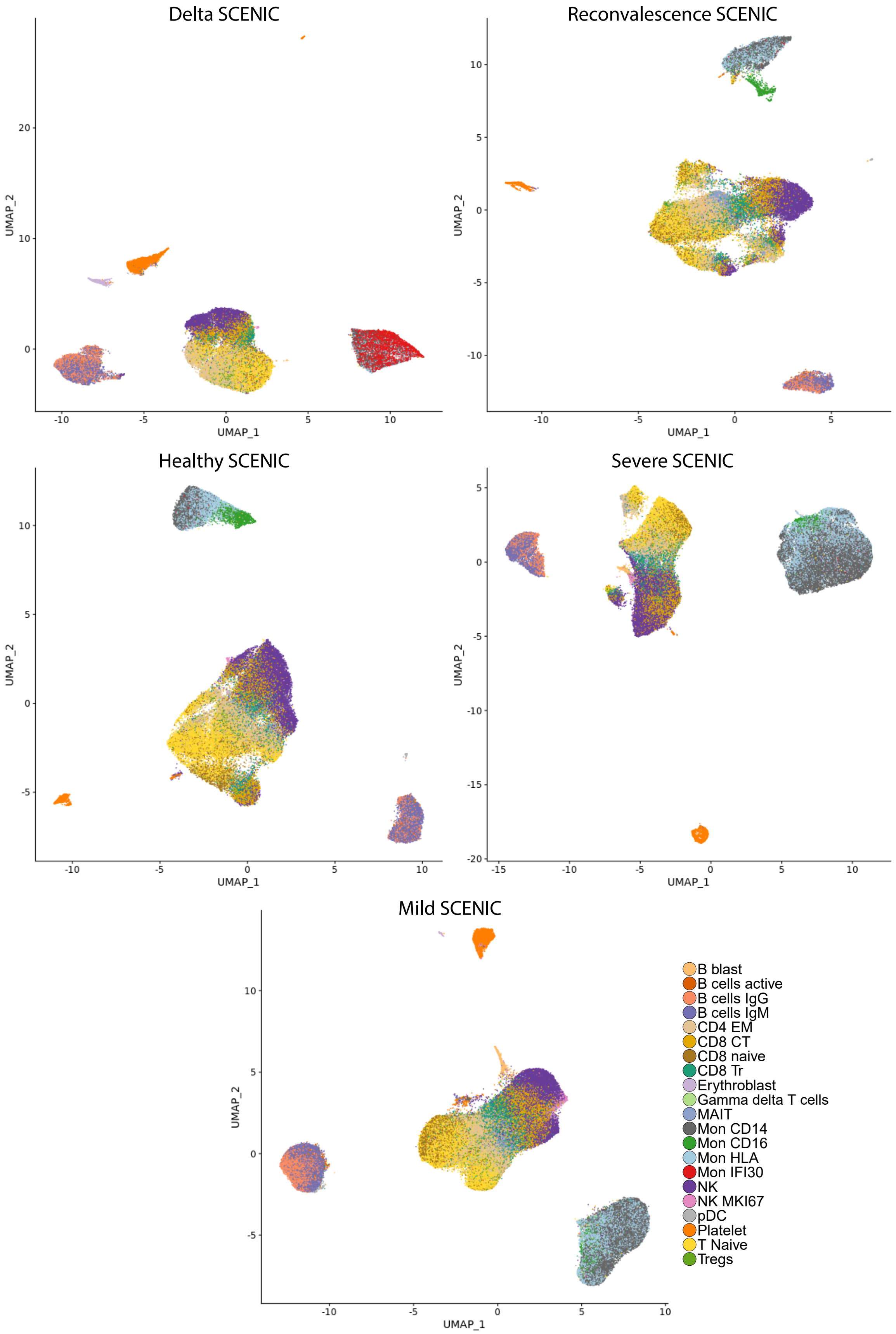

Figure S9. SCENIC UMAP plots for individually processed cohortes. Cell types are colored based on the Seurat CCA integration. Annotation was projected on the UMAP obtained for SCENIC cell clustering performed for each cohorte independently. Mon IFI30 (shon in red) were identified as a cluster only in severe Delta cases.

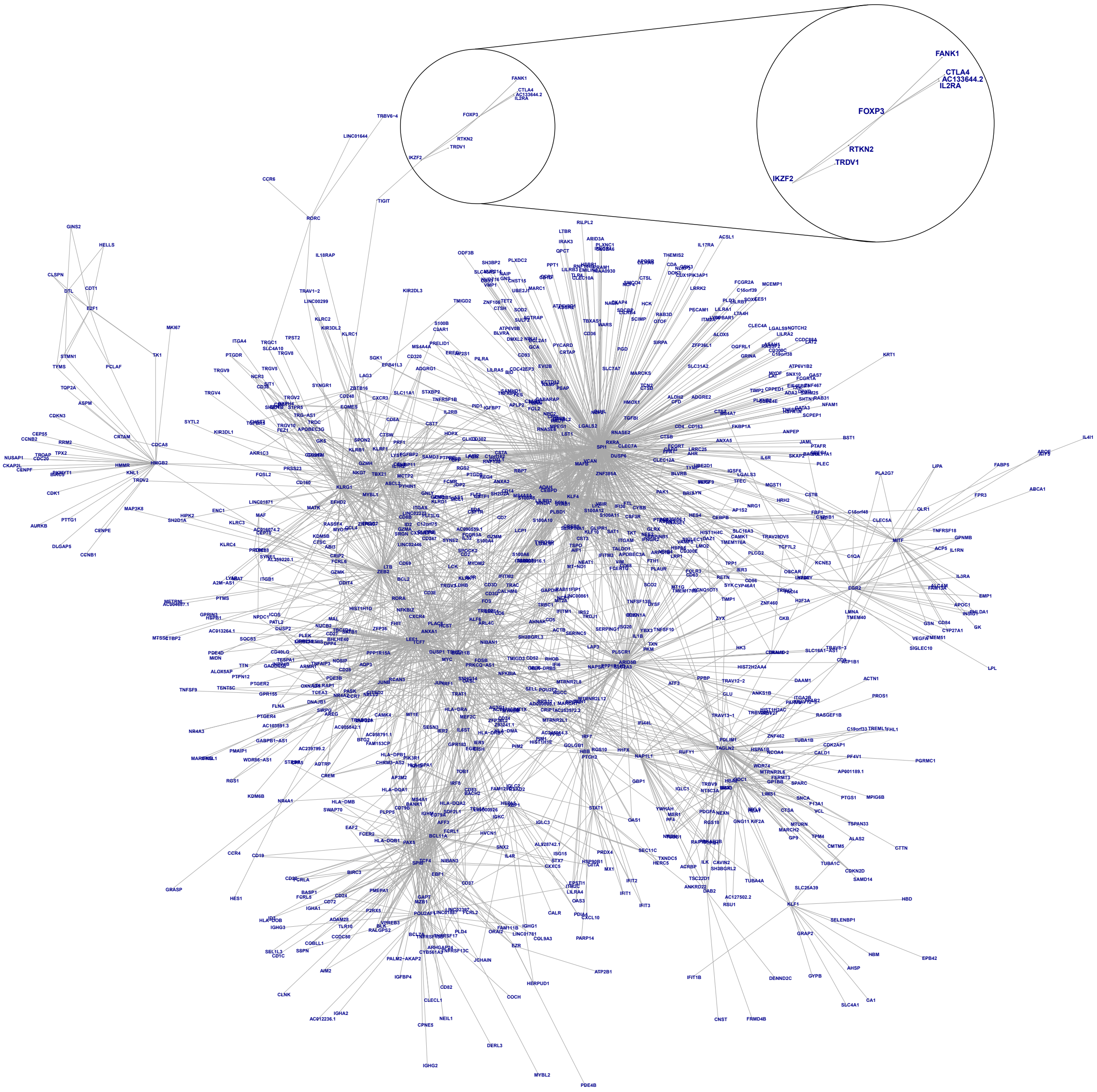

Figure S10. Entire gene regulatory network. Gene regulatory network inferred using SCENIC. In order to obtain a strong biological signal we keep only genes with importance scores above 99th percentile. FOXP3 with target genes are highlighted to demonstrate that SCENIC GRN captures known regulators and targets of the minor Treg population in PBMC.

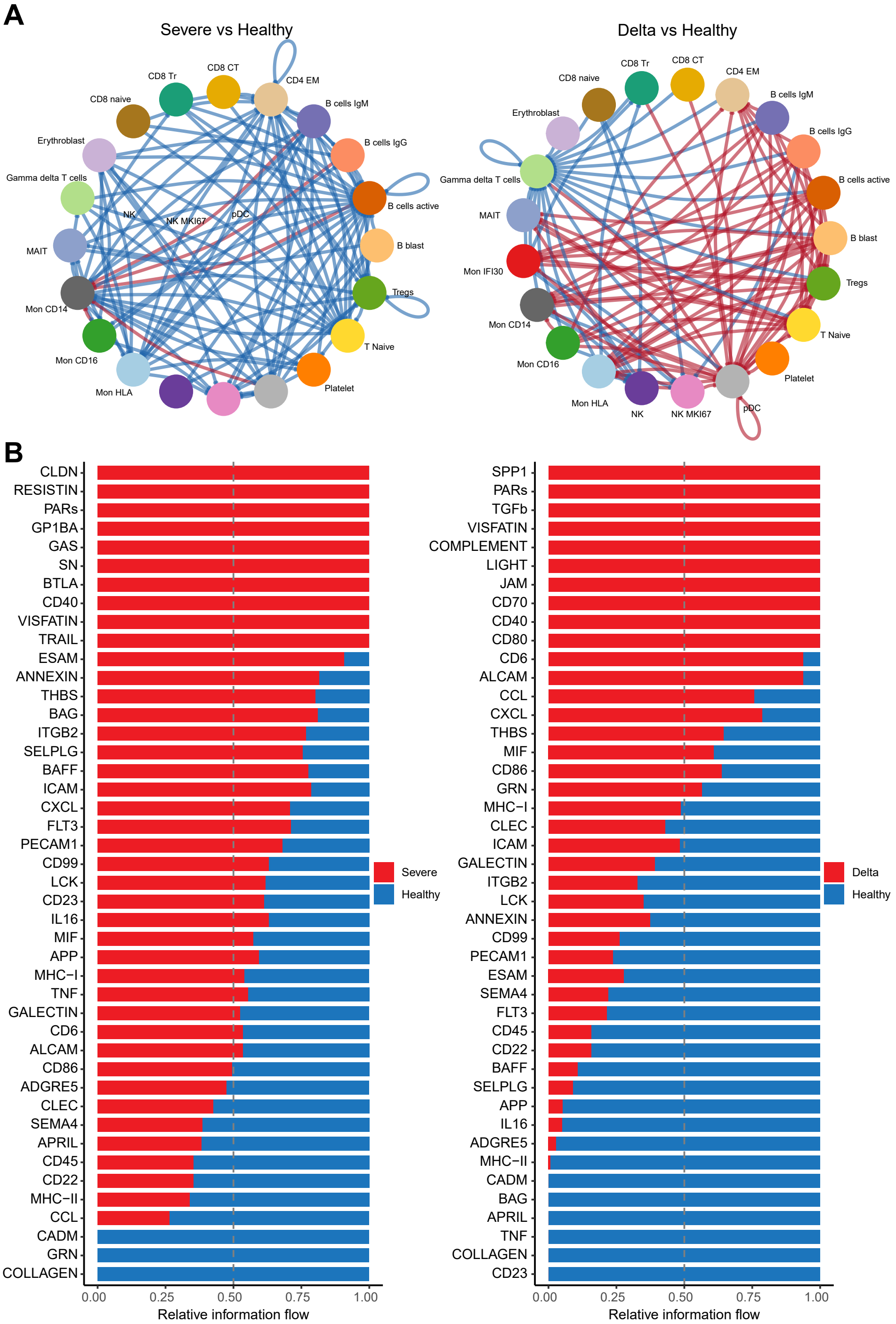

Figure S11. Cellchat differential interactions. (**A**) Circle plot showing predicted differential cell-cell communications between severe Wuhan-like and healthy groups on the left and between Delta and healthy groups on the right. Red lines indicate gained and blue lost cell-cell communications (**B**) Barplots showing the intensity of context-specific signalling pathways measured as relative information flow as implemented in CellChat. TGF beta and SPP1 are predicted as main signalling molecules involved in cell-cell communication for severe Delta COVID-19 in comparison with healthy individuals. For the severe Wuhan-like samples Resistin, and Caledon were predicted as main communication molecules.

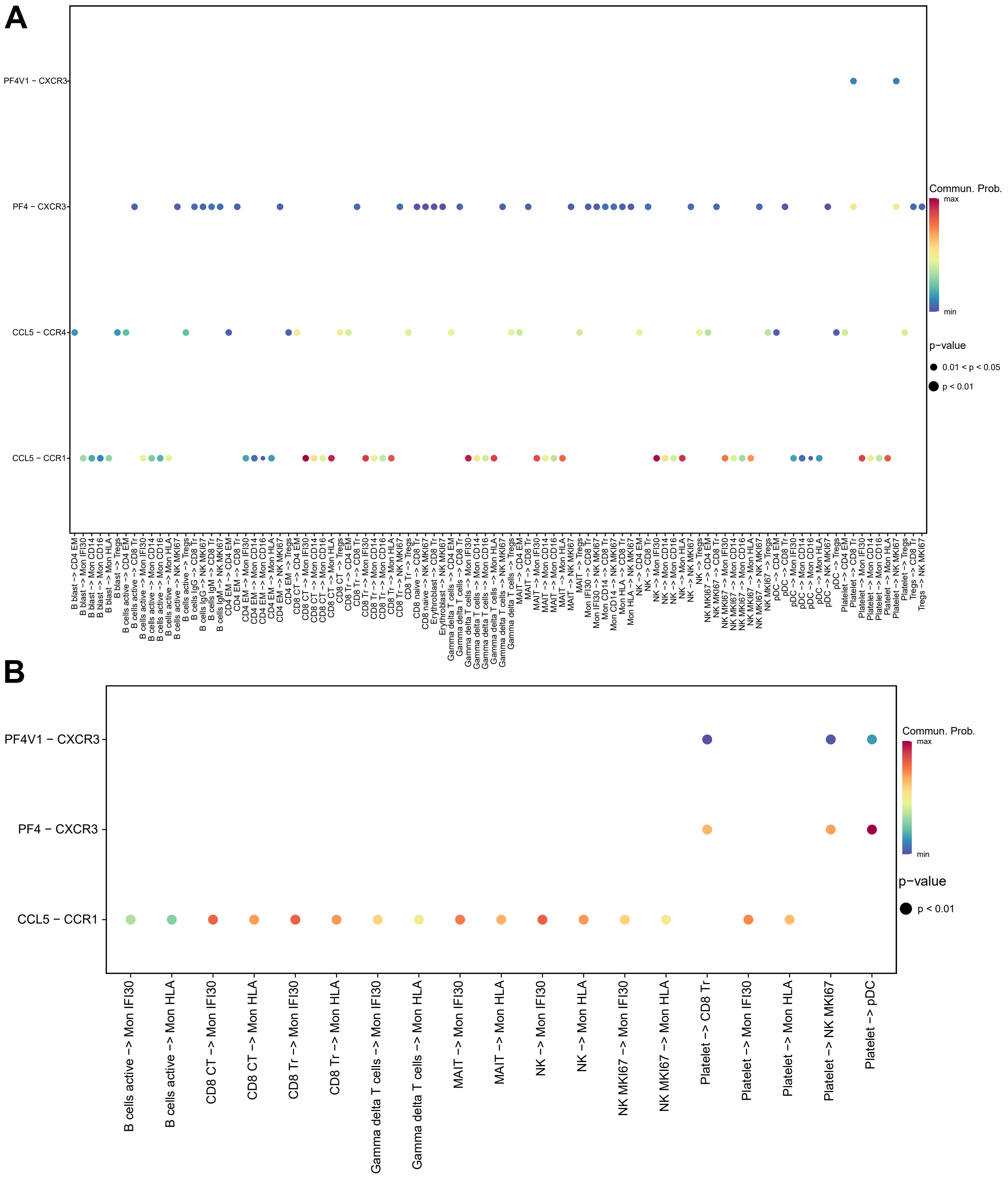

Figure S12. Dotplot for communication molecules of the CCL-CXCL pathway. (**A**) Dotplot showing cell pairs (x-axis) and predicted interacting ligand-receptors (y-axis) for CCL-CXCL pathways obtained for severe Wuhan-like groups. Colour on the dotplot shows the strength of a given ligand-receptor interaction quantitatively represented by a probability value obtained with CellChat using law of mass action and approximated based on the average expression of the ligand-receptors by query cell groups. (**B**) Dotplot showing cell pairs (x-axis) and predicted interacting ligand-receptors (y-axis) for CCL-CXCL pathways obtained for severe Delta group. Colour on the dotplot shows the strength of a given ligand-receptor interaction quantitatively represented by a probability value obtained with CellChat using law of mass action and approximated based on the average expression of the ligand-receptors by query cell groups.

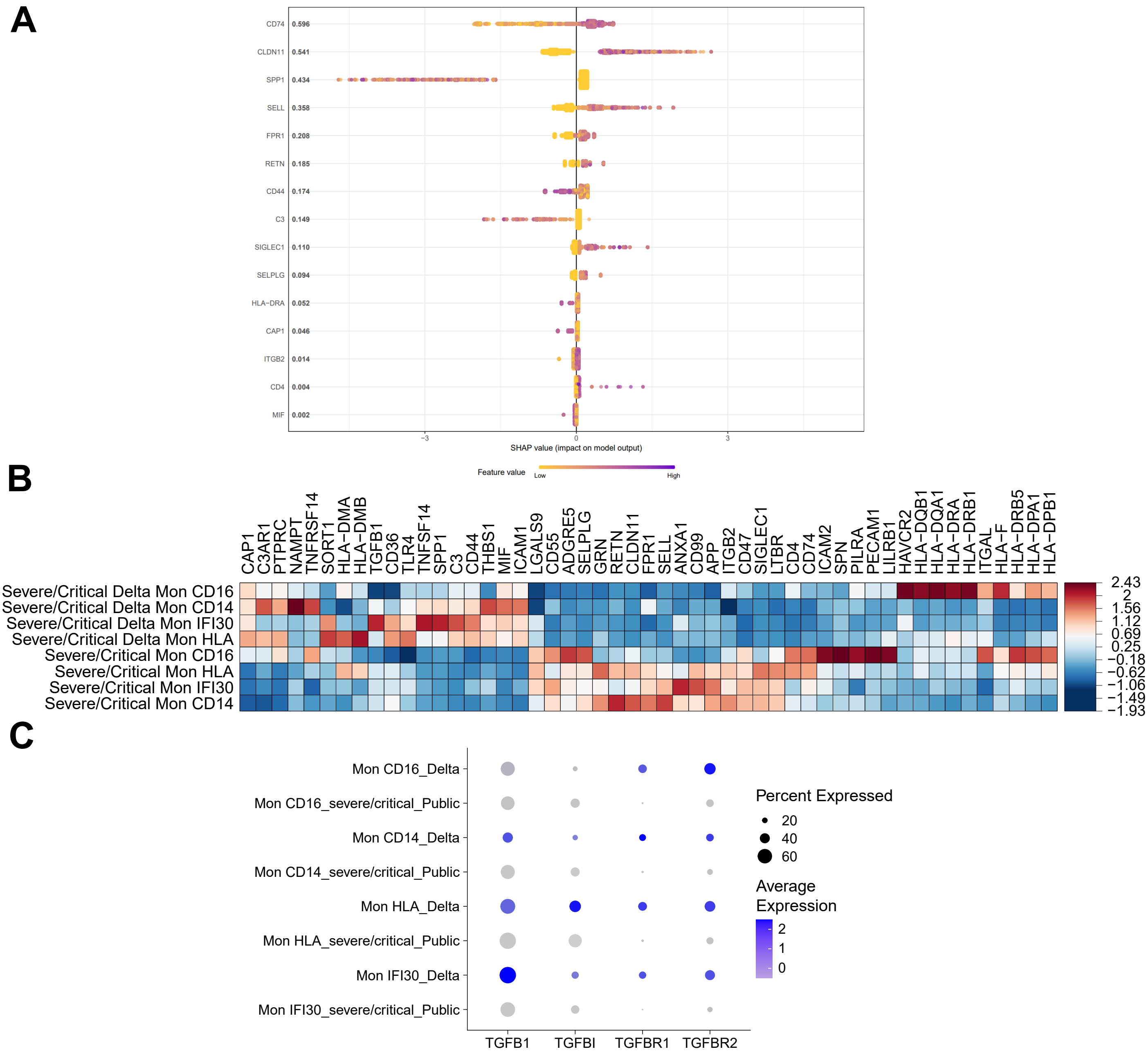

Figure S13. Ligand and receptors contrasting severe Delta and Wuhan-like cases based on the gene expression in monocytes. (**A**) Heatmap with SHAP (SHapley Additive exPlanations) values indicating feature contribution to a classification decision. XGboost binary classifiers have been trained to contrast monocytes. Features are normalised gene expression of the ligand and receptors (from CellChatDB), and cohort (severe Delta or Wuhan-like variant) are class labels. (**B**) Heatmap with average expression of the ligands and receptors in monocyte subtypes from severe Delta and Wuhan-like samples. Monocytes from the same disease cohort cluster with each other more than with common cell type monocytes that indicate about global shift in ligand-receptor interactions brought by virus variant. (**C**) Expression of the TGFB1, TGFBI, TGFBR1, TGFBR2 in monocytes for severe Delta and Wuhan-like samples. All types of monocytes from samples with Delta variant show higher level of the TGF beta related molecules.

Table S1. Description of the in-house and public datasets used in the study

SampleID	Severity	Dataset	Data_source
108	Rec	Reconvalescence	In-house
109	Healthy	Healthy	In-house
110	Healthy	Healthy	In-house
112	Healthy	Healthy	In-house
113	Healthy	Healthy	In-house
114	Healthy	Healthy	In-house
115	Healthy	Healthy	In-house
134	mild/moderate	Mild/Moderate	In-house
144	mild/moderate	Mild/Moderate	In-house
147	mild/moderate	Mild/Moderate	In-house
148	mild/moderate	Mild/Moderate	In-house
151	mild/moderate	Mild/Moderate	In-house
165	Healthy	Healthy	In-house
166	Healthy	Healthy	In-house
167	Rec	Reconvalescence	In-house
172	Rec	Reconvalescence	In-house
207	Healthy	Healthy	In-house
208	Rec	Reconvalescence	In-house
218	Rec	Reconvalescence	In-house
245	Rec	Reconvalescence	In-house
3	severe/critical	Flu	Public (GSE149689)
4	severe/critical	Flu	Public (GSE149689)
413	mild/moderate	Mild/Moderate	In-house
414	mild/moderate	Mild/Moderate	In-house
415	mild/moderate	Mild/Moderate	In-house
416	mild/moderate	Mild/Moderate	In-house
417	mild/moderate	Mild/Moderate	In-house
418	mild/moderate	Mild/Moderate	In-house
419	mild/moderate	Mild/Moderate	In-house
420	mild/moderate	Mild/Moderate	In-house
561	severe/critical	Severe/Critical Delta	In-house
562	severe/critical	Severe/Critical Delta	In-house
564	severe/critical	Severe/Critical Delta	In-house
565	severe/critical	Severe/Critical Delta	In-house
567	severe/critical	Severe/Critical Delta	In-house
568	severe/critical	Severe/Critical Delta	In-house
569	severe/critical	Severe/Critical Delta	In-house
573	severe/critical	Severe/Critical Delta	In-house
574	severe/critical	Severe/Critical Delta	In-house
6	severe/critical	Flu	Public (GSE149689)
67	mild/moderate	Mild/Moderate	In-house
68	mild/moderate	Mild/Moderate	In-house
69	mild/moderate	Mild/Moderate	In-house
7	severe/critical	Flu	Public (GSE149689)
8	severe/critical	Flu	Public (GSE149689)
S-M017	mild/moderate	Mild/Moderate	Public (GSE158055)
S-M023	mild/moderate	Mild/Moderate	Public (GSE158055)
S-M035-1	mild/moderate	Mild/Moderate	Public (GSE158055)
S-M041-2	mild/moderate	Mild/Moderate	Public (GSE158055)
S-M043-1	mild/moderate	Mild/Moderate	Public (GSE158055)
S-M043-2	mild/moderate	Mild/Moderate	Public (GSE158055)
S-M044-1	mild/moderate	Mild/Moderate	Public (GSE158055)
S-M046	mild/moderate	Mild/Moderate	Public (GSE158055)
S-M047	mild/moderate	Mild/Moderate	Public (GSE158055)
S-M065	mild/moderate	Mild/Moderate	Public (GSE158055)
S-M067	mild/moderate	Mild/Moderate	Public (GSE158055)
S-M069	mild/moderate	Mild/Moderate	Public (GSE158055)
S-M073	mild/moderate	Mild/Moderate	Public (GSE158055)
S-M074-2	mild/moderate	Mild/Moderate	Public (GSE158055)
S-M076-2	mild/moderate	Mild/Moderate	Public (GSE158055)
S-M078	mild/moderate	Mild/Moderate	Public (GSE158055)
S-S001-2	severe/critical	Severe/Critical	Public (GSE158055)
S-S028	severe/critical	Severe/Critical	Public (GSE158055)
S-S029	severe/critical	Severe/Critical	Public (GSE158055)
S-S030	severe/critical	Severe/Critical	Public (GSE158055)
S-S031	severe/critical	Severe/Critical	Public (GSE158055)
S-S054	severe/critical	Severe/Critical	Public (GSE158055)
S-S057	severe/critical	Severe/Critical	Public (GSE158055)
S-S077	severe/critical	Severe/Critical	Public (GSE158055)
S-S079	severe/critical	Severe/Critical	Public (GSE158055)
S-S082	severe/critical	Severe/Critical	Public (GSE158055)
S-S085-2	severe/critical	Severe/Critical	Public (GSE158055)
S-S086-2	severe/critical	Severe/Critical	Public (GSE158055)
S-S089-2	severe/critical	Severe/Critical	Public (GSE158055)
S-S090-2	severe/critical	Severe/Critical	Public (GSE158055)
S-S091	severe/critical	Severe/Critical	Public (GSE158055)
S-S092	severe/critical	Severe/Critical	Public (GSE158055)
