# Supplementary figures and images for: Single-Cell Gene Expression Analysis Revealed Immune Cell Signatures of Delta COVID-19
Source: Cells. 2022 Sep 21;11(19):2950. doi: 10.3390/cells11192950 (PMC9563974; doi:10.3390/cells11192950)

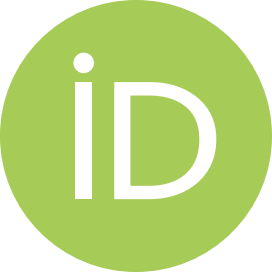

Supplement: Supplementary file 1 [file cells-11-02950-s001.zip › Definitions/logo-orcid.pdf]

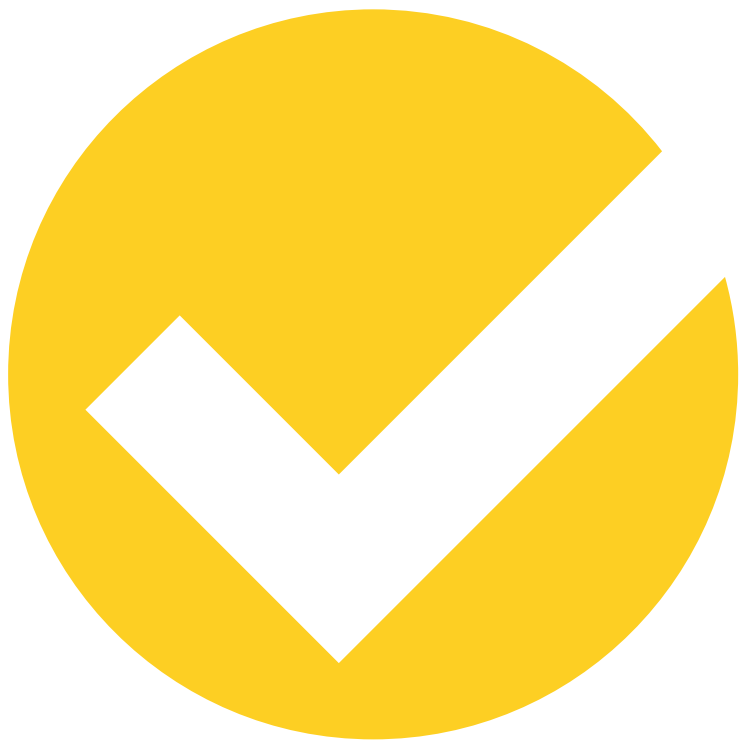

check for  
updates

Supplement: Supplementary file 1 [file cells-11-02950-s001.zip › Definitions/logo-updates.pdf]

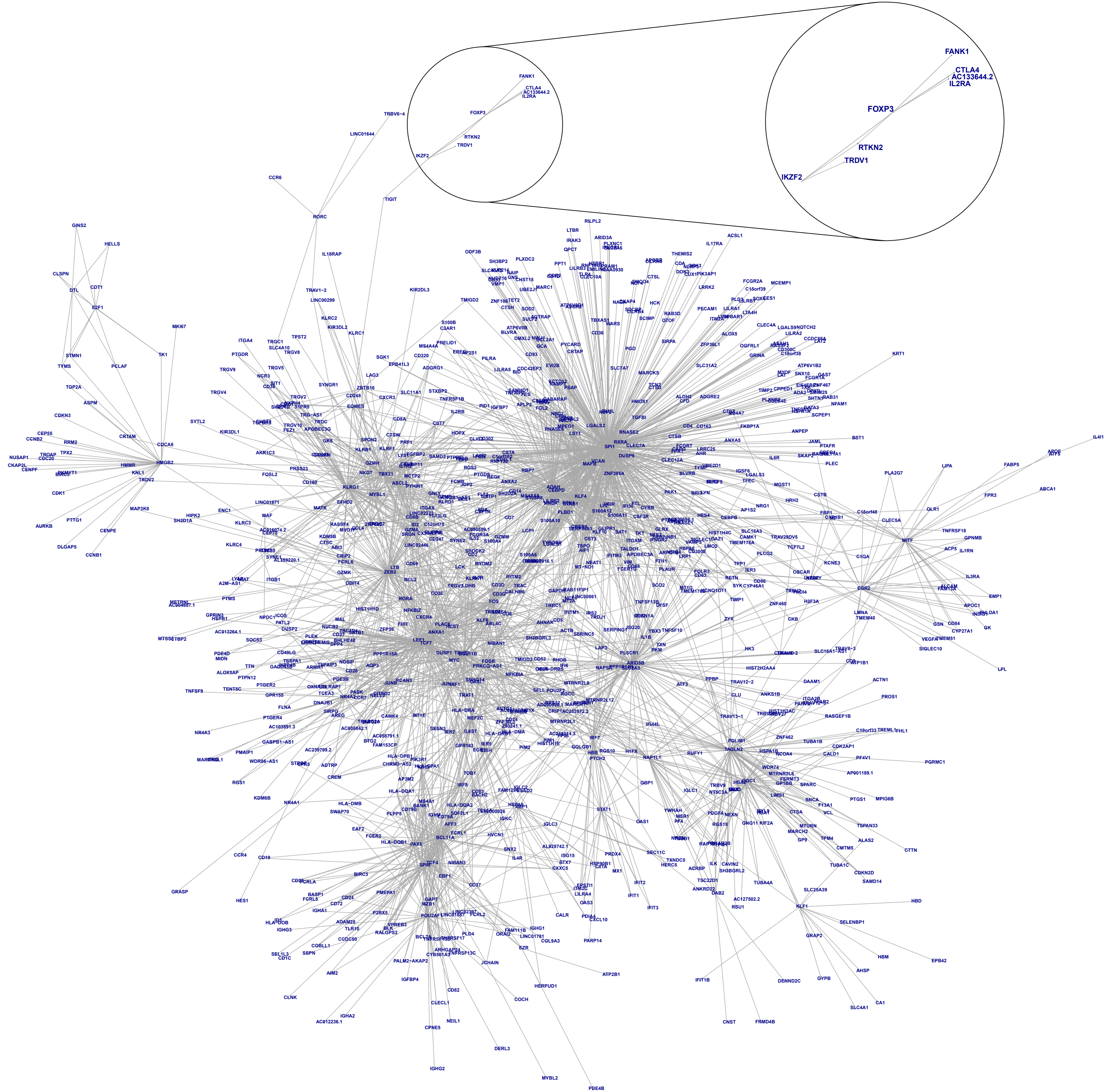

Supplement: Supplementary file 1 [file cells-11-02950-s001.zip › supfiles/S11.pdf]

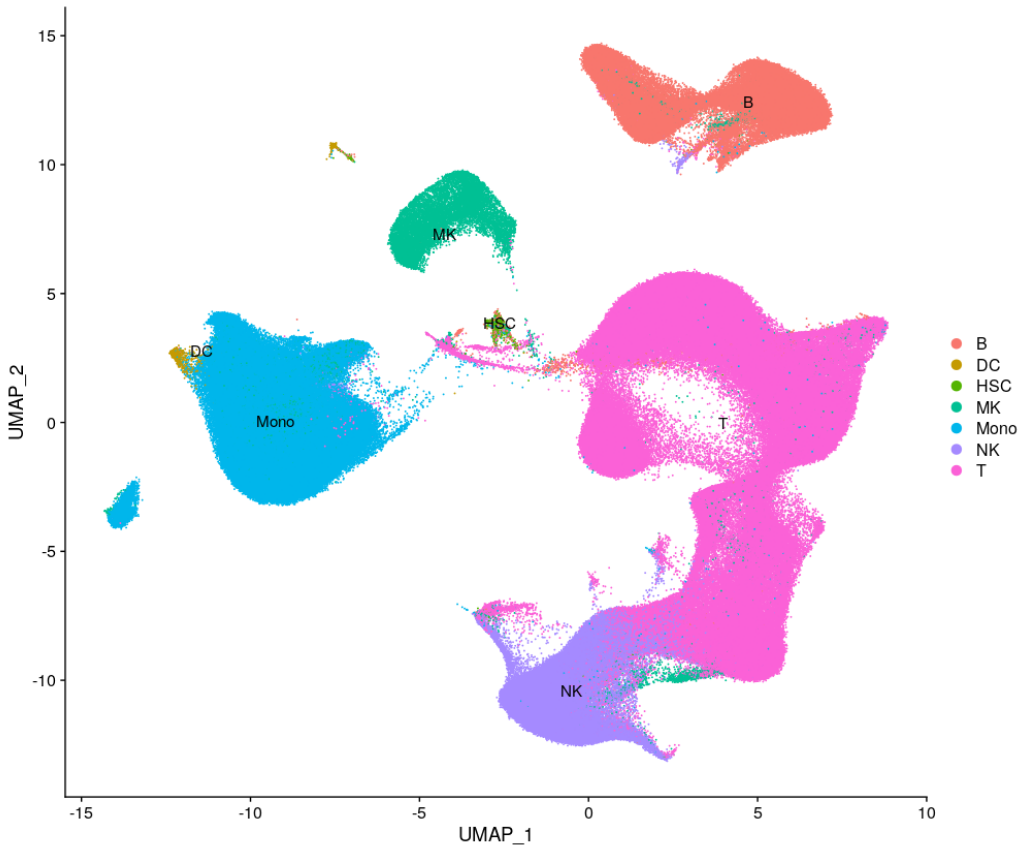

Supplement: Supplementary file 1 [file cells-11-02950-s001.zip › supfiles/S14.png]

Split Identity

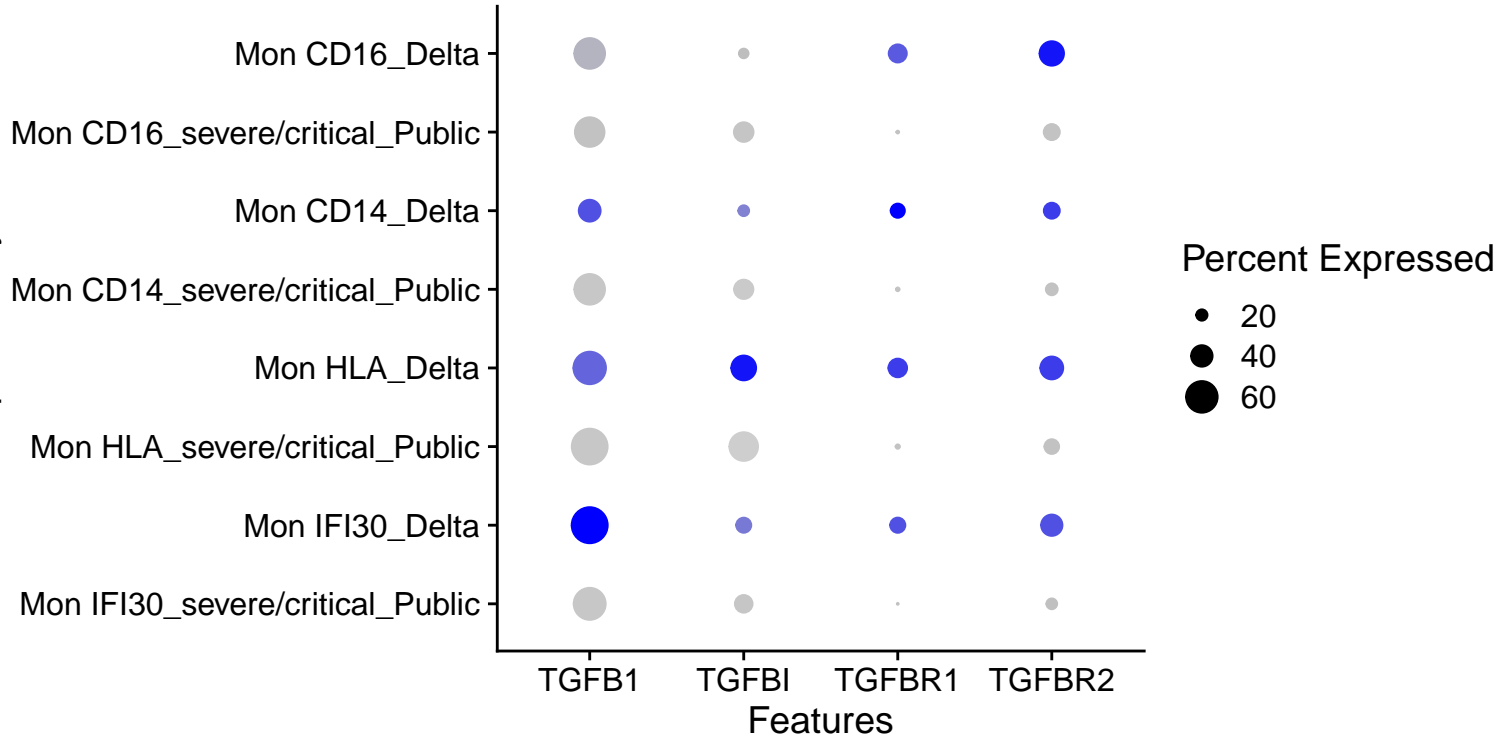

Supplement: Supplementary file 1 [file cells-11-02950-s001.zip › supfiles/S12.pdf]

NK MKI67

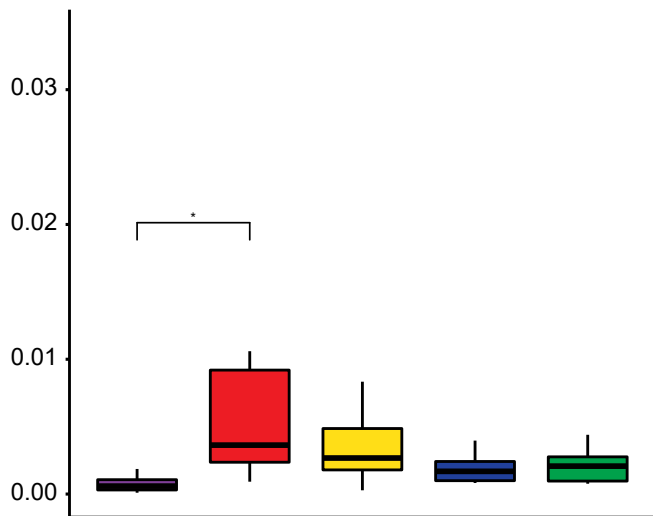

pDC

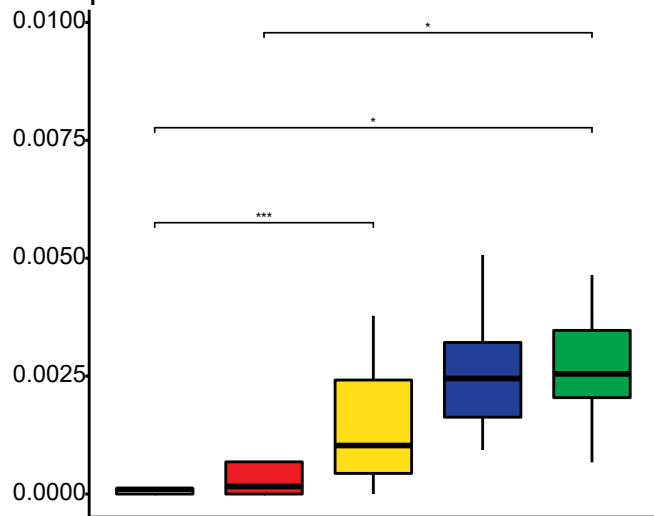

B blast

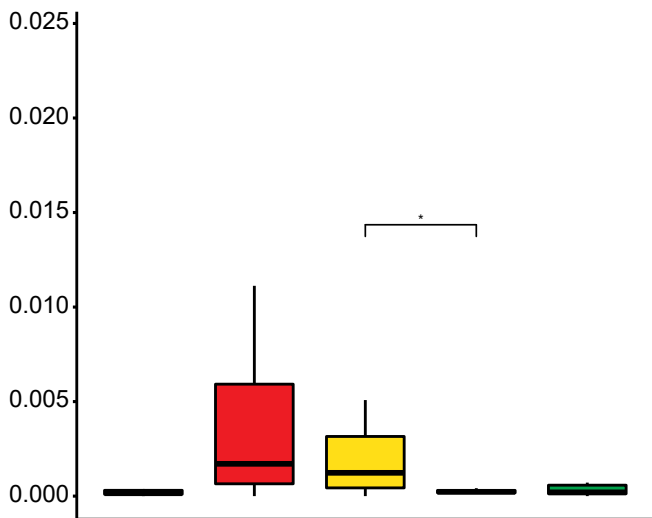

B cells active

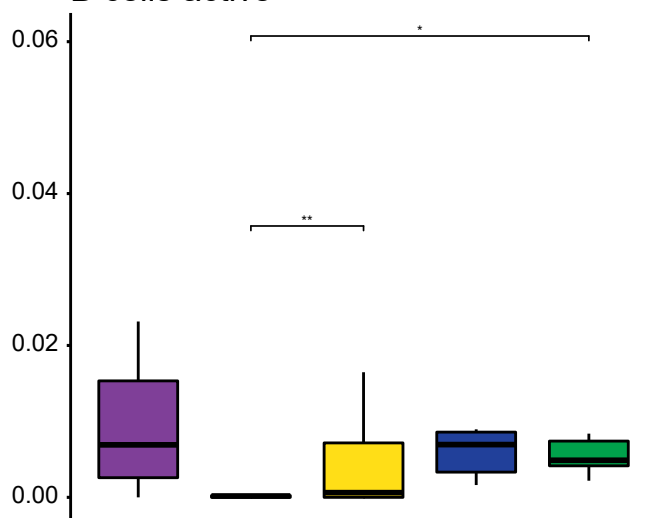

CD8 naive

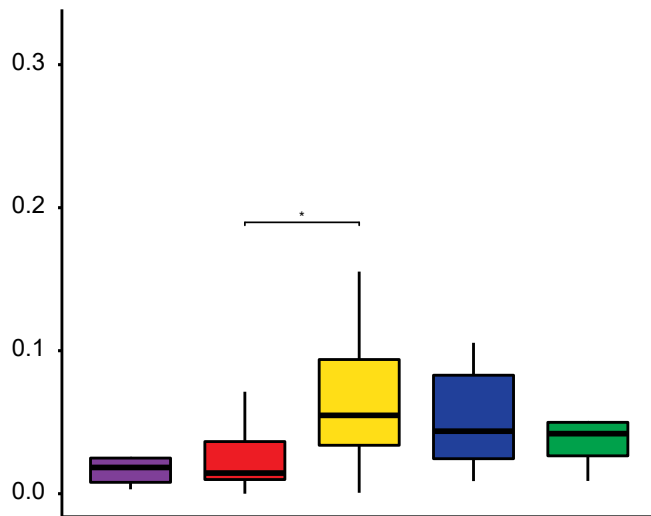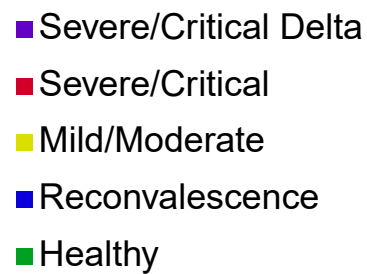

Supplement: Supplementary file 1 [file cells-11-02950-s001.zip › supfiles/S3 copy.pdf]

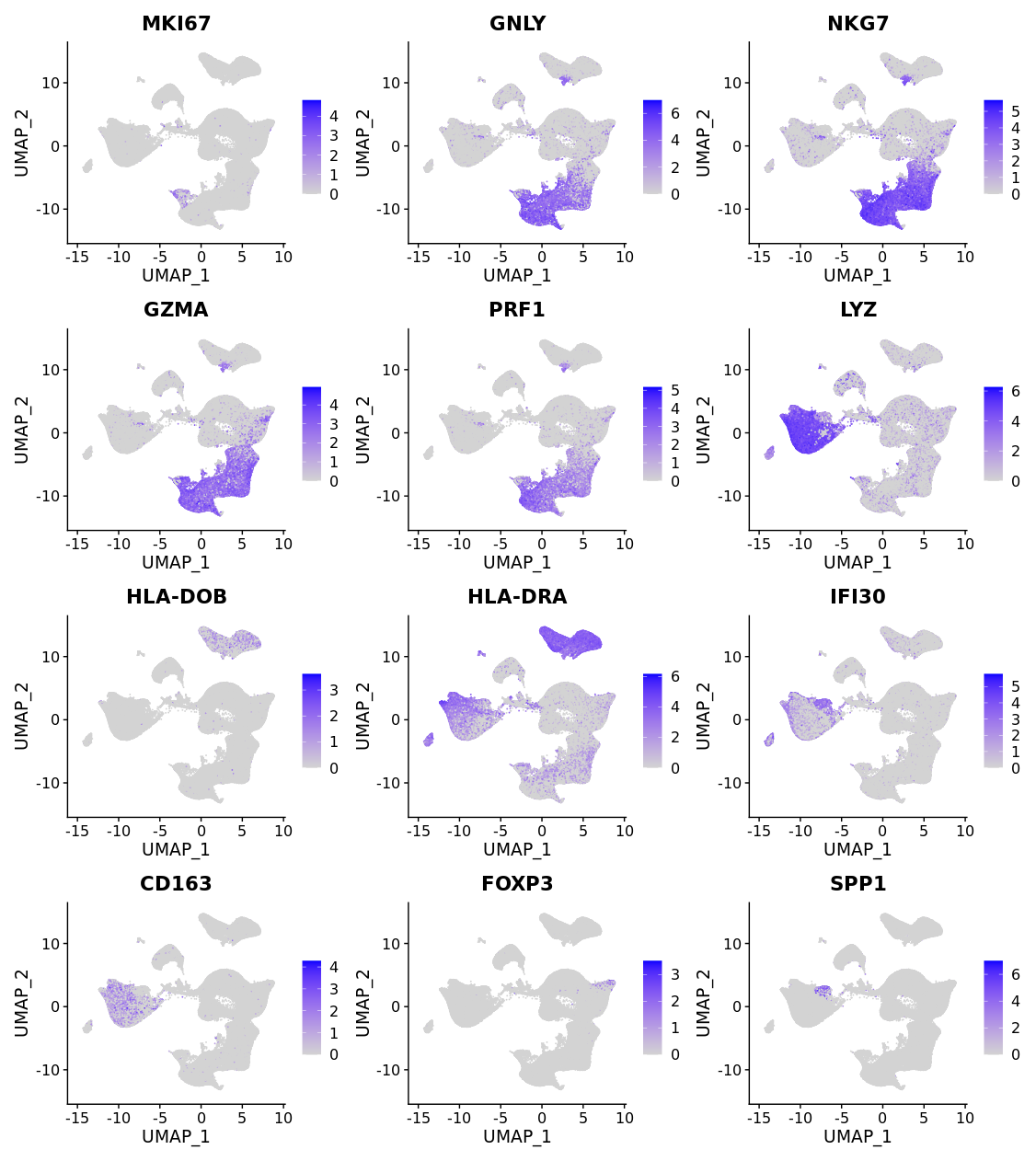

Supplement: Supplementary file 1 [file cells-11-02950-s001.zip › supfiles/S2.png]

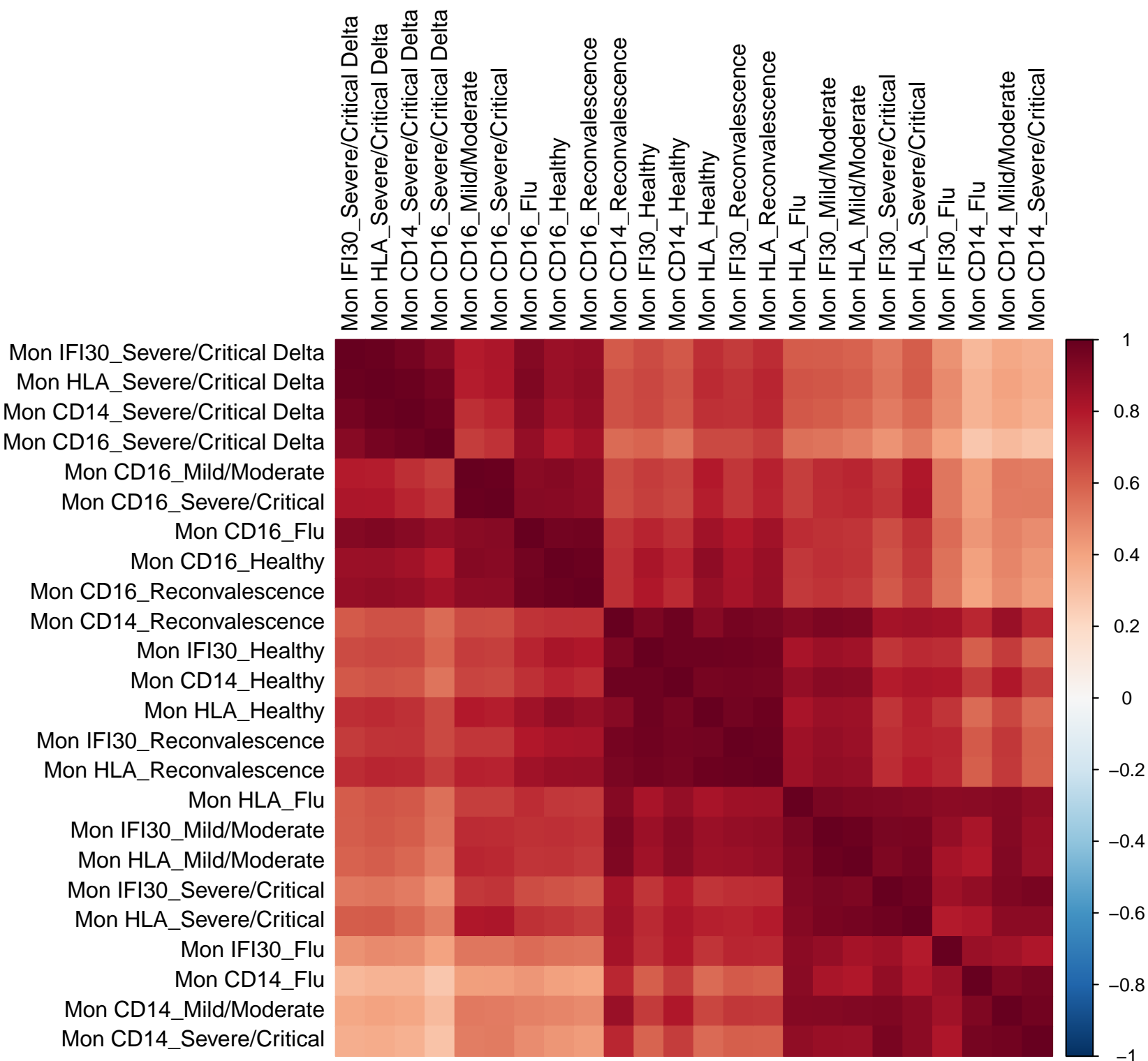

Supplement: Supplementary file 1 [file cells-11-02950-s001.zip › supfiles/S13.pdf]

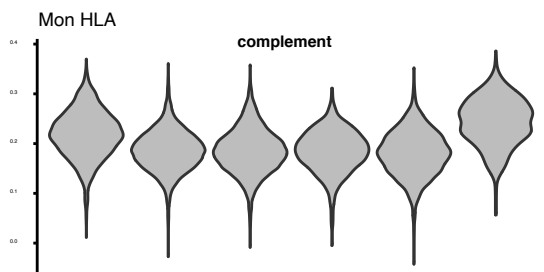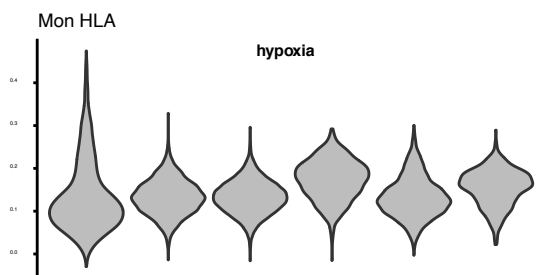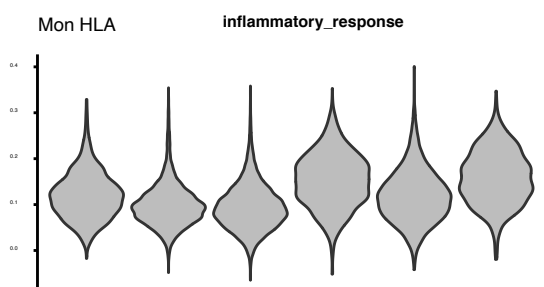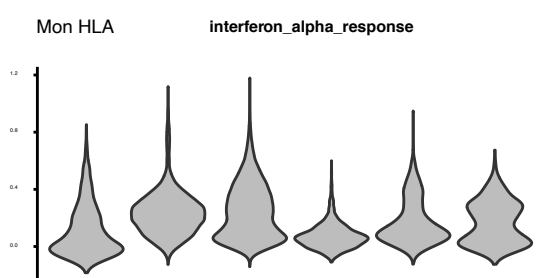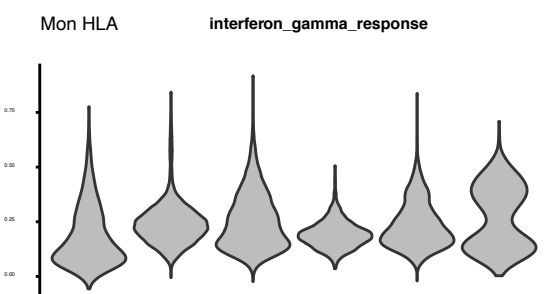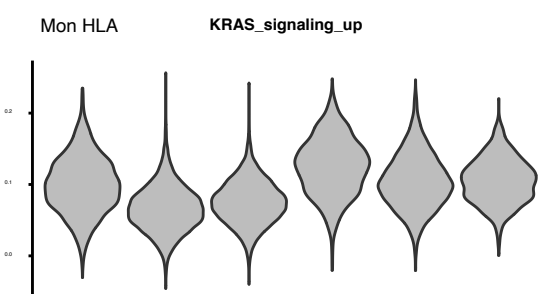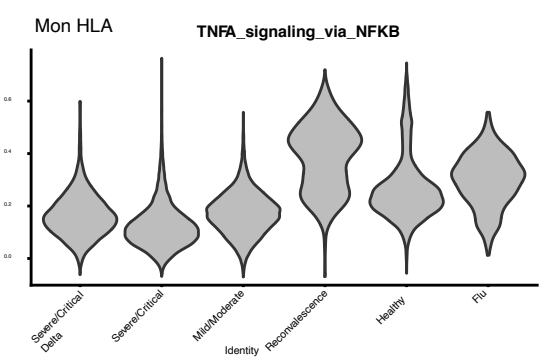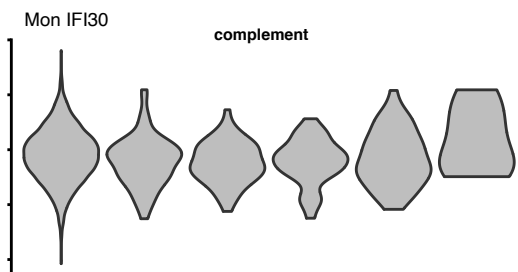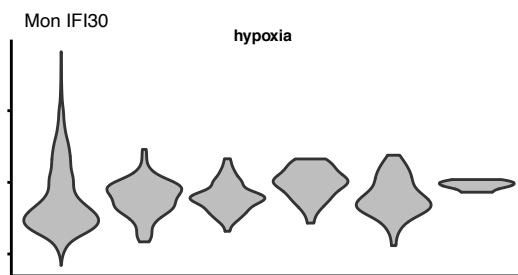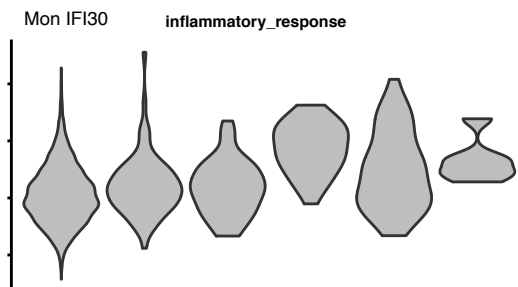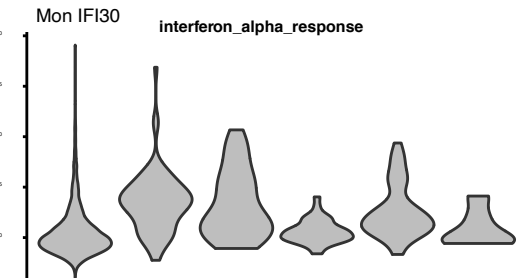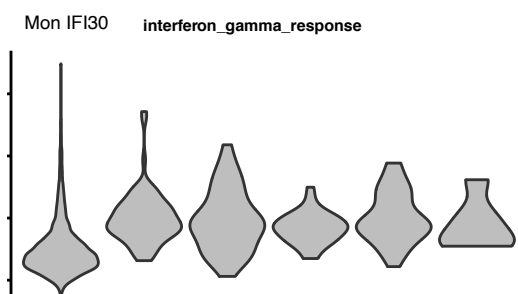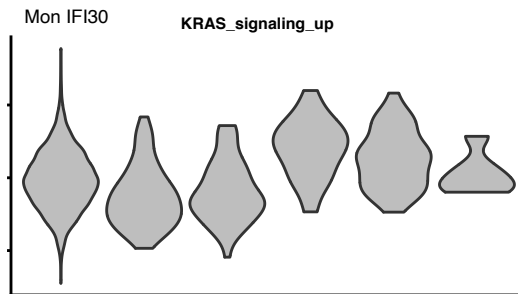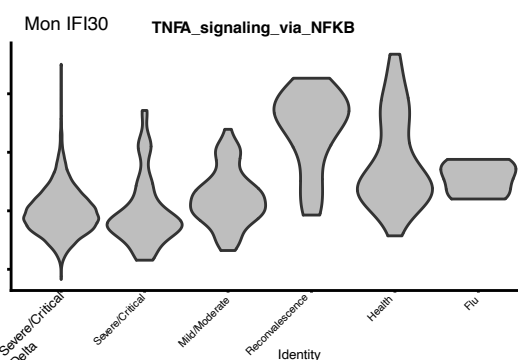

Supplement: Supplementary file 1 [file cells-11-02950-s001.zip › supfiles/supp4_final.pdf]

Severe vs Healthy

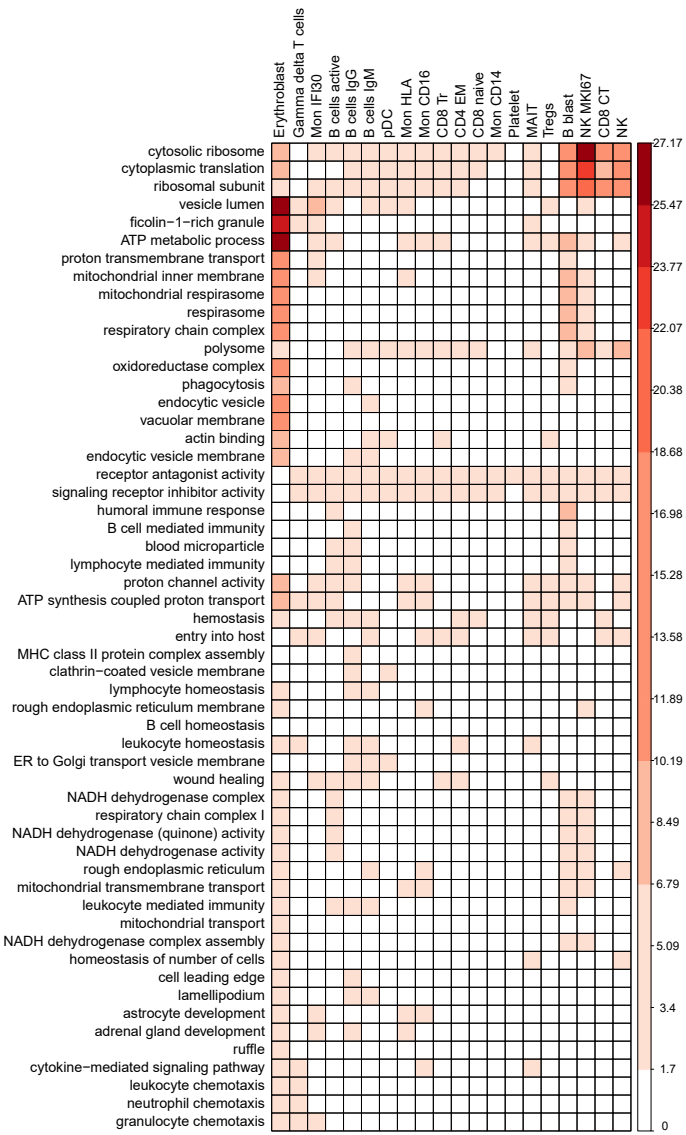

Delta vs Healthy

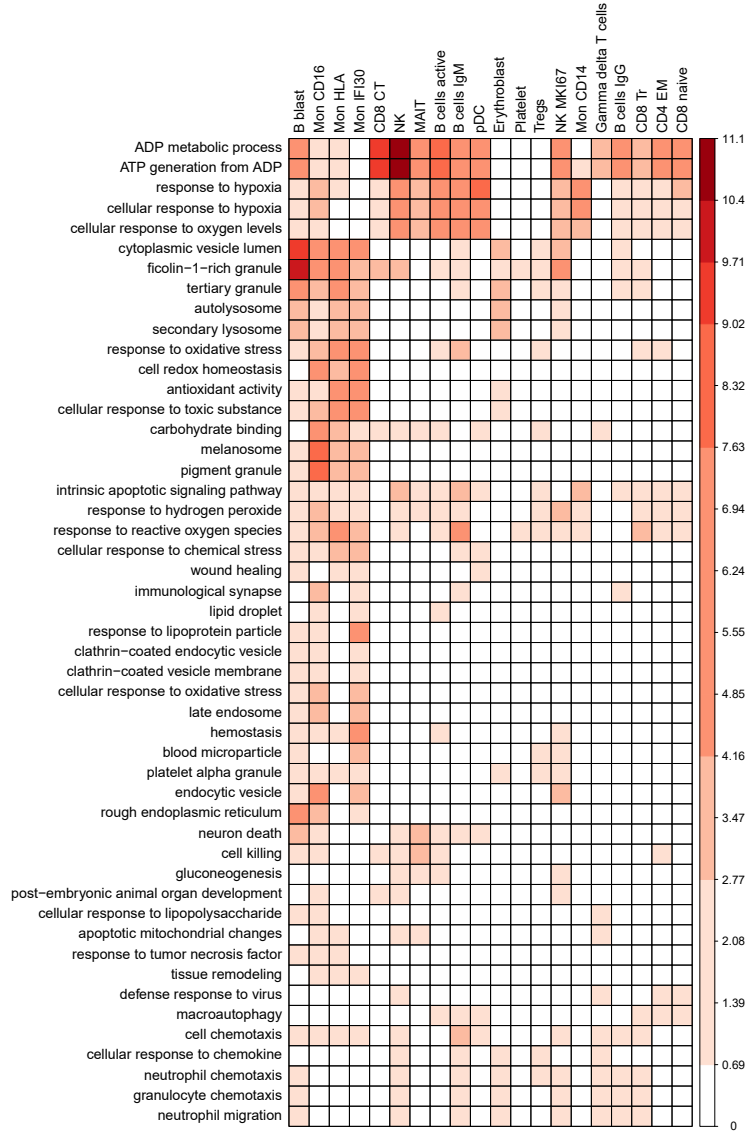

Supplement: Supplementary file 1 [file cells-11-02950-s001.zip › supfiles/S9 copy.pdf]

**A**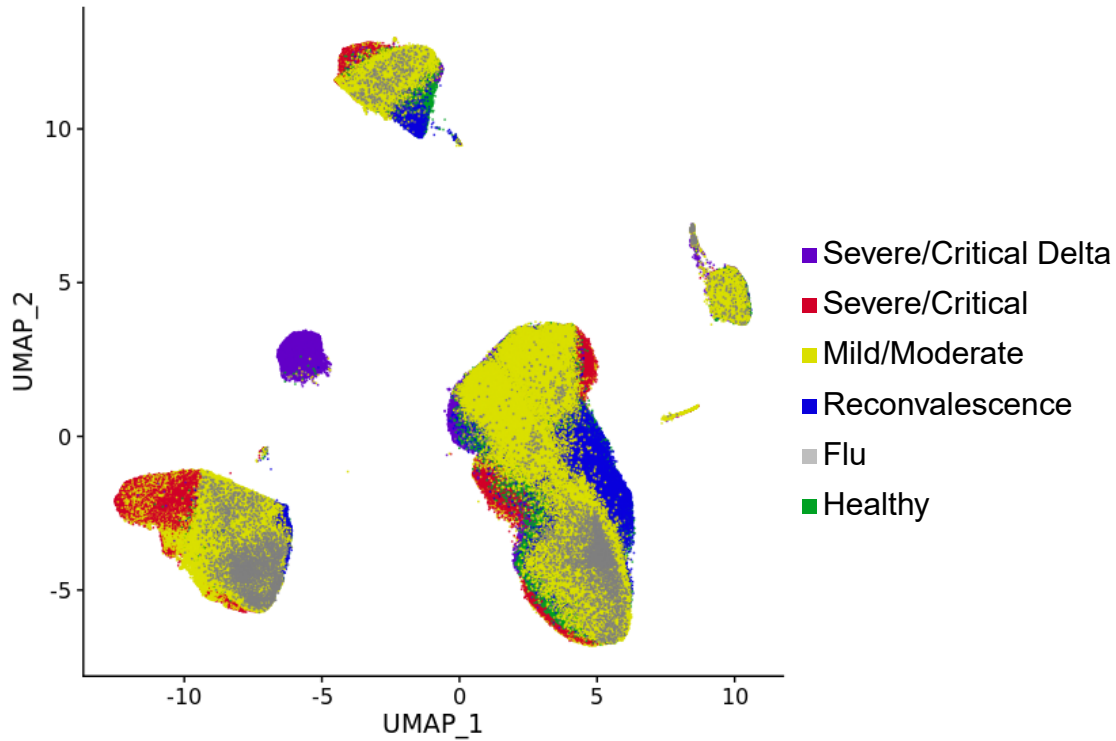**B**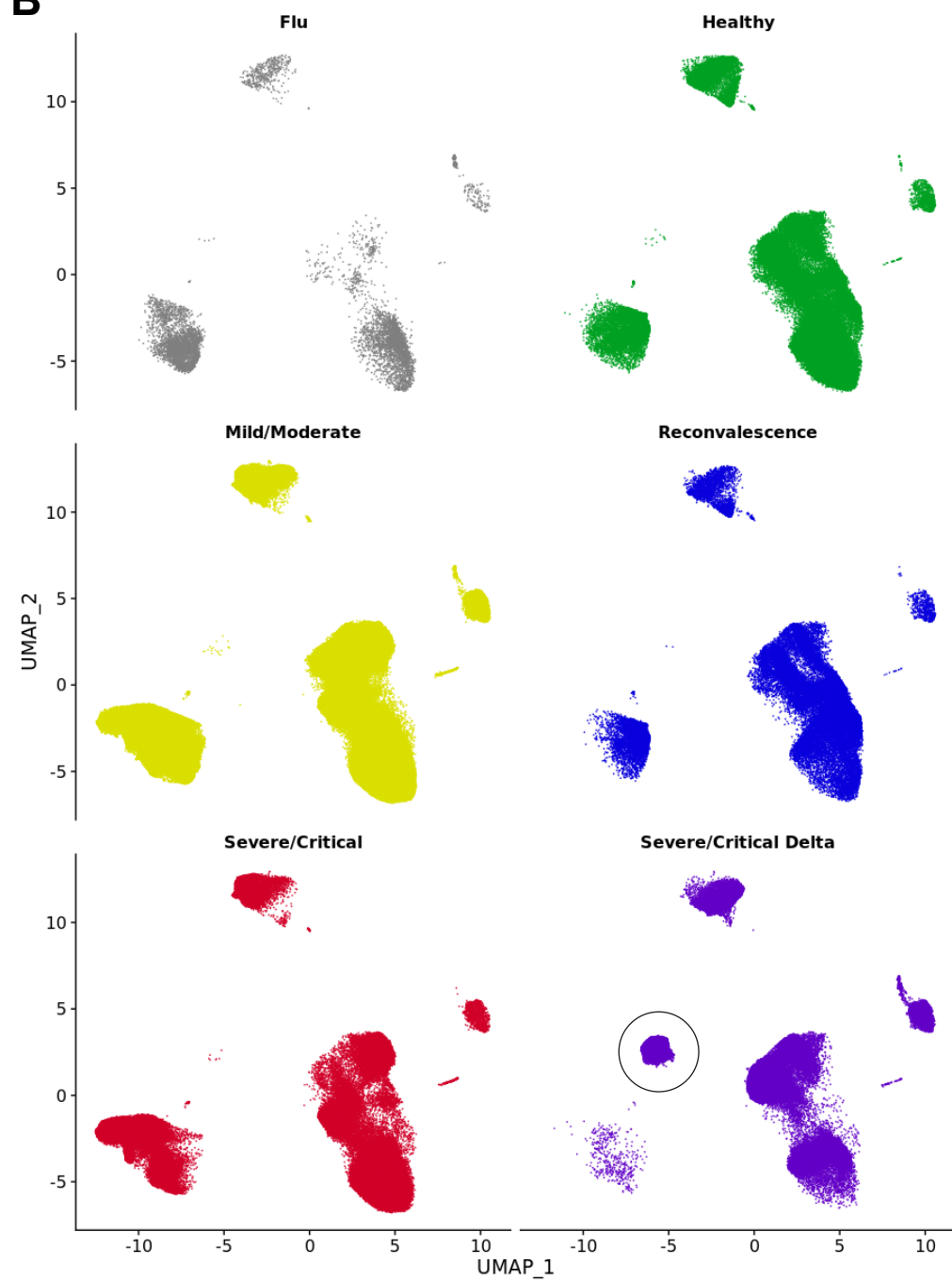

Supplement: Supplementary file 1 [file cells-11-02950-s001.zip › supfiles/S7 copy.pdf]

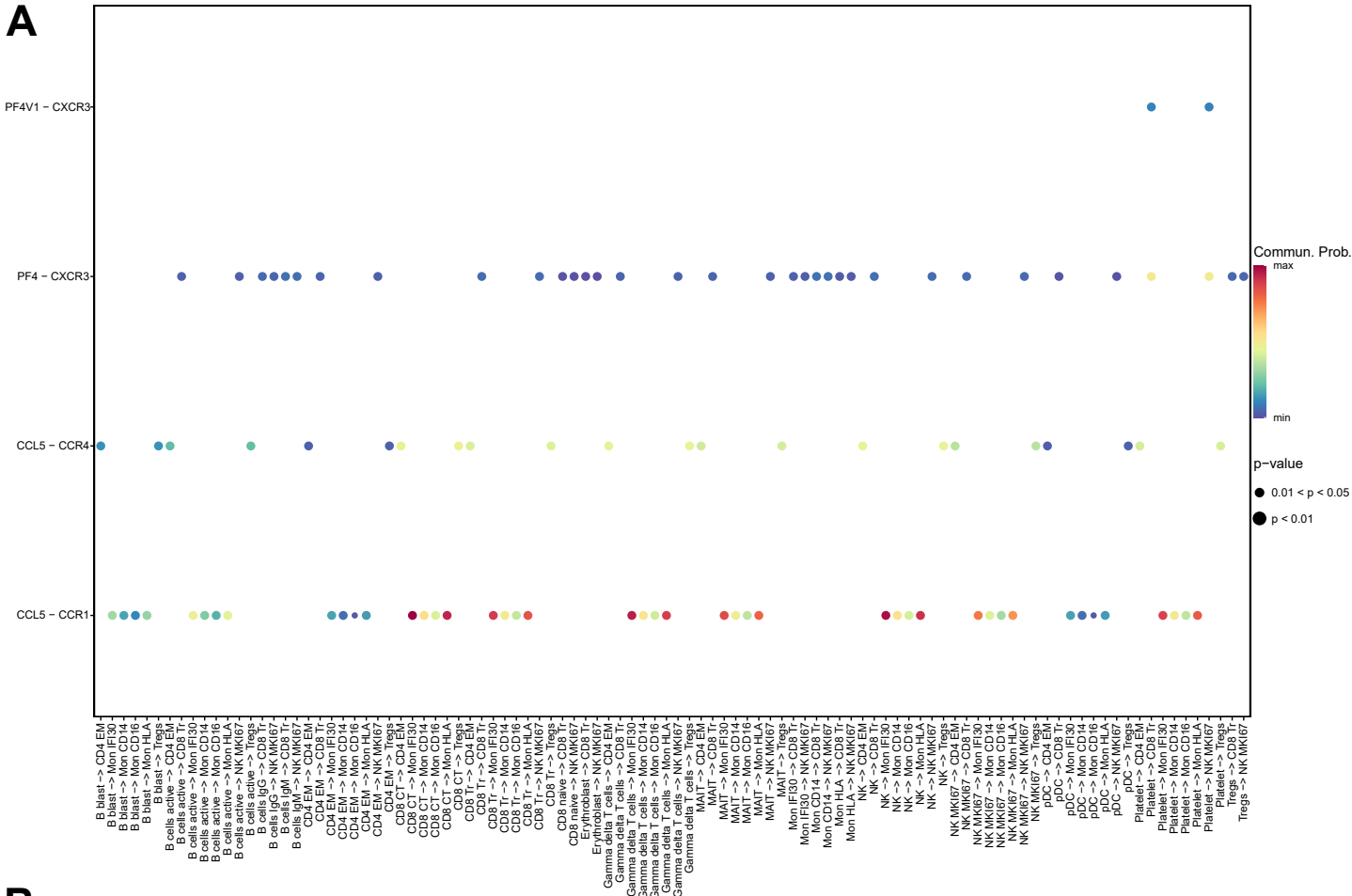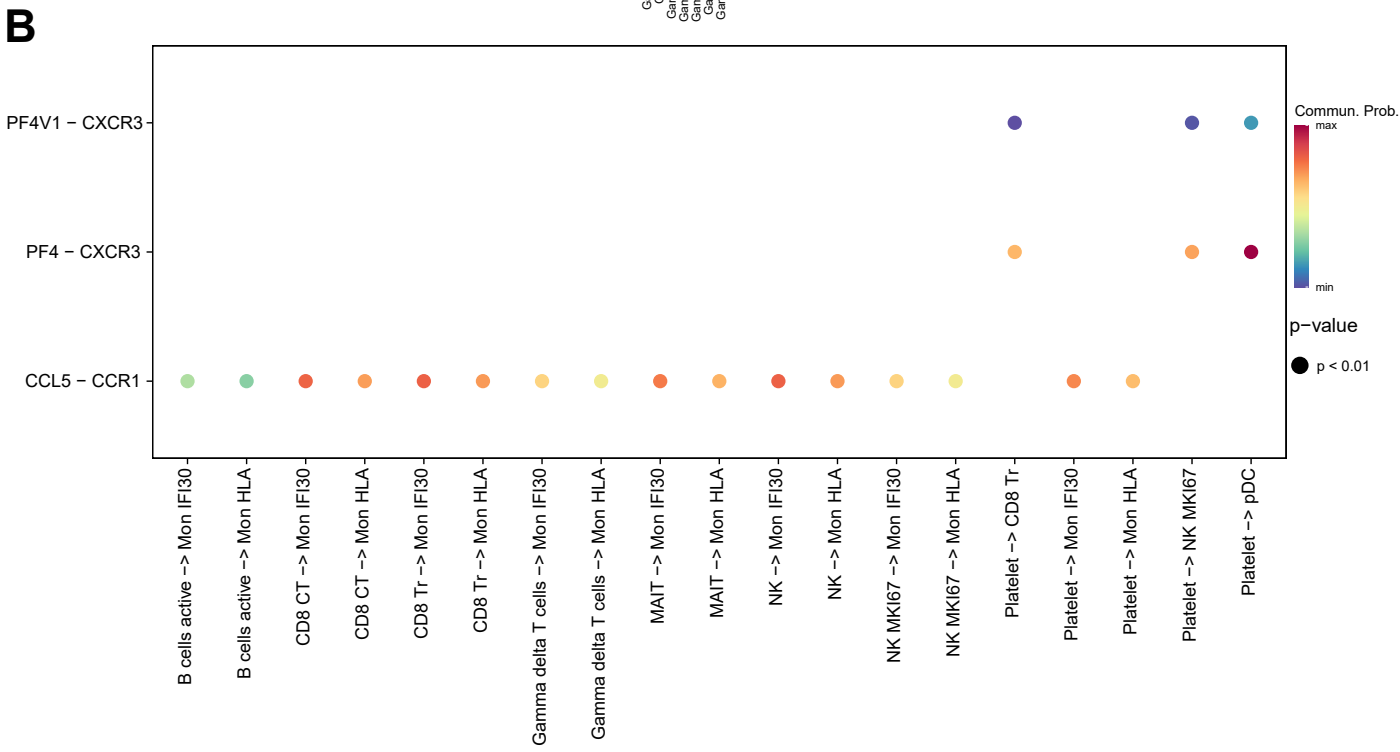

Supplement: Supplementary file 1 [file cells-11-02950-s001.zip › supfiles/S10 copy.pdf]

**A**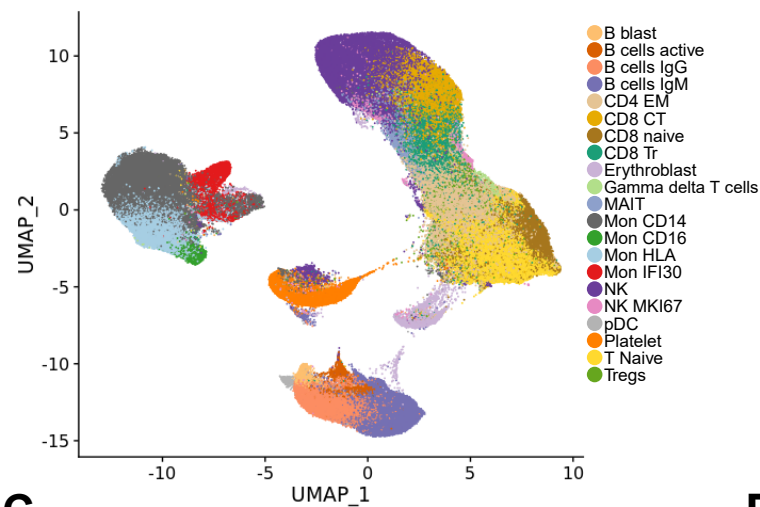**B**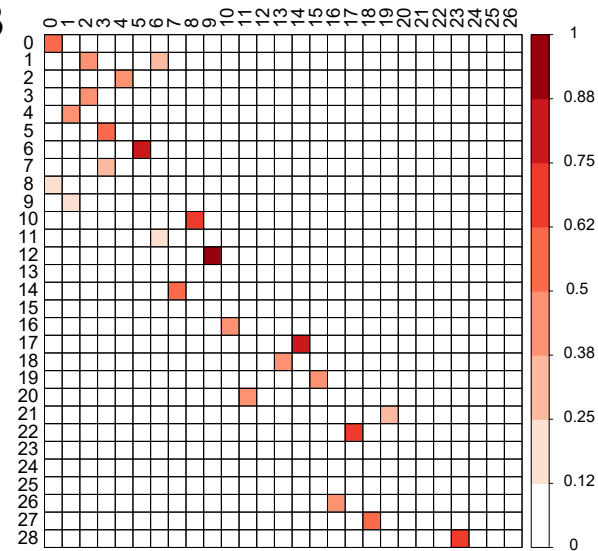**C**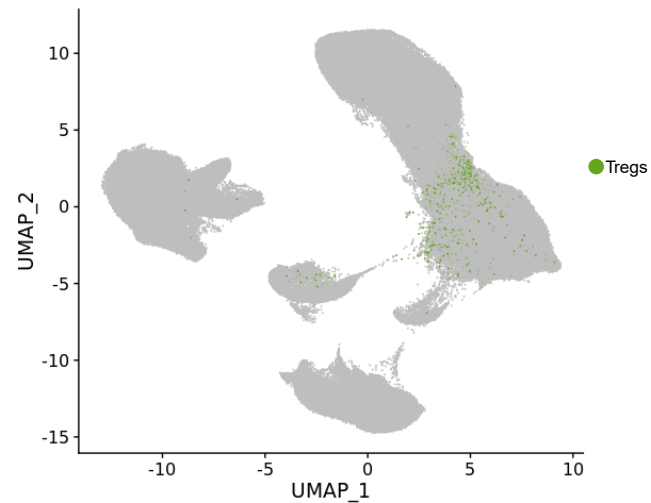**D**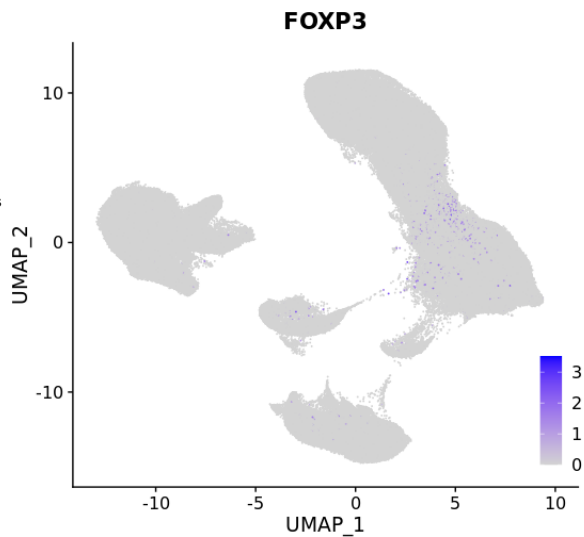

Supplement: Supplementary file 1 [file cells-11-02950-s001.zip › supfiles/S1 copy.pdf]

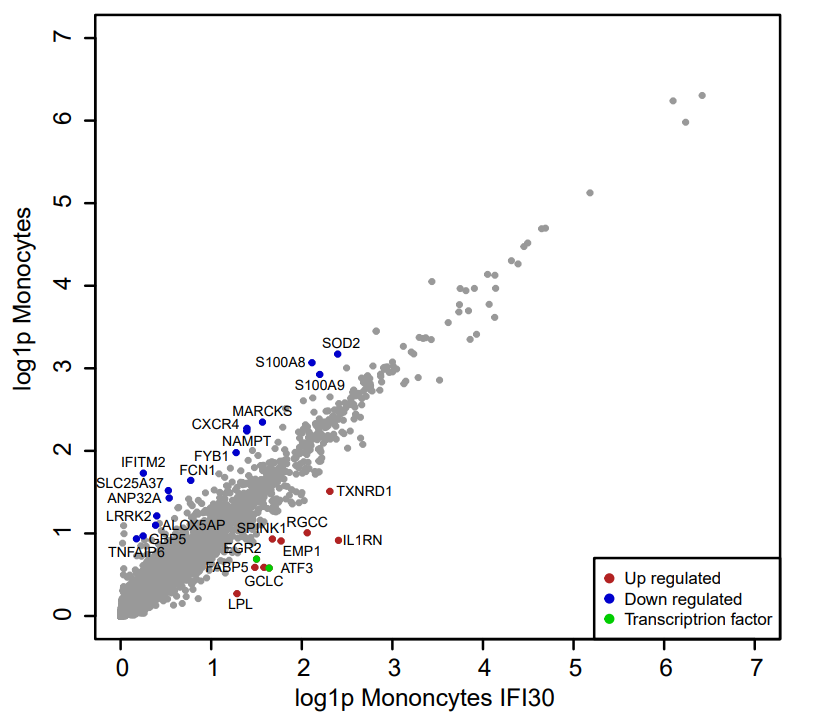

Supplement: Supplementary file 1 [file cells-11-02950-s001.zip › supfiles/sup_scatter.png]

A

## Severe vs Healthy

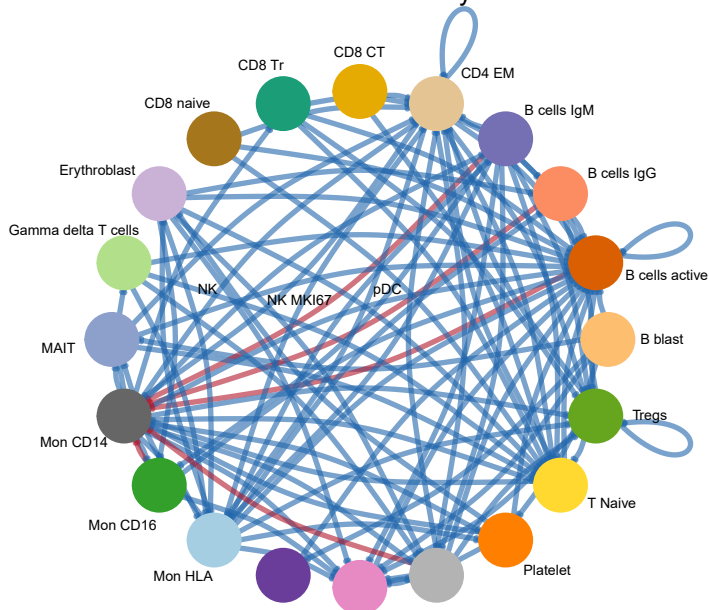

## Delta vs Healthy

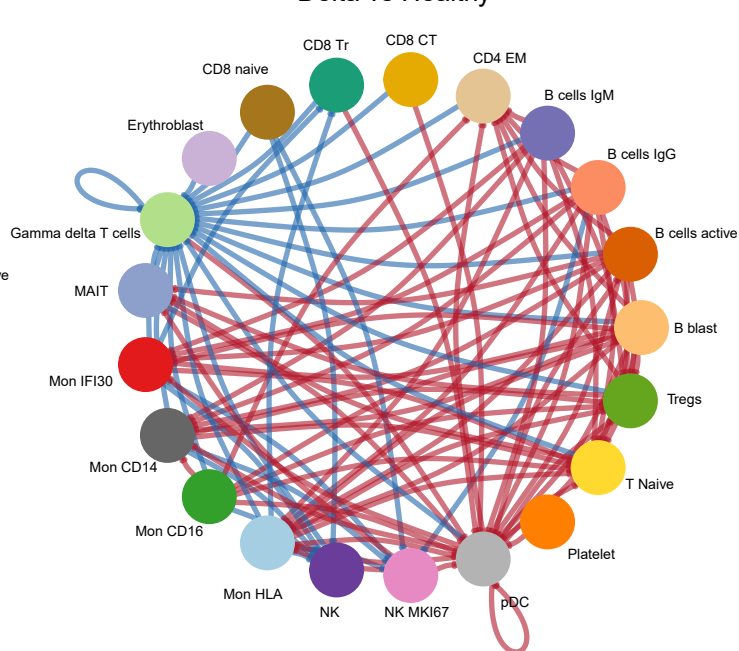

B

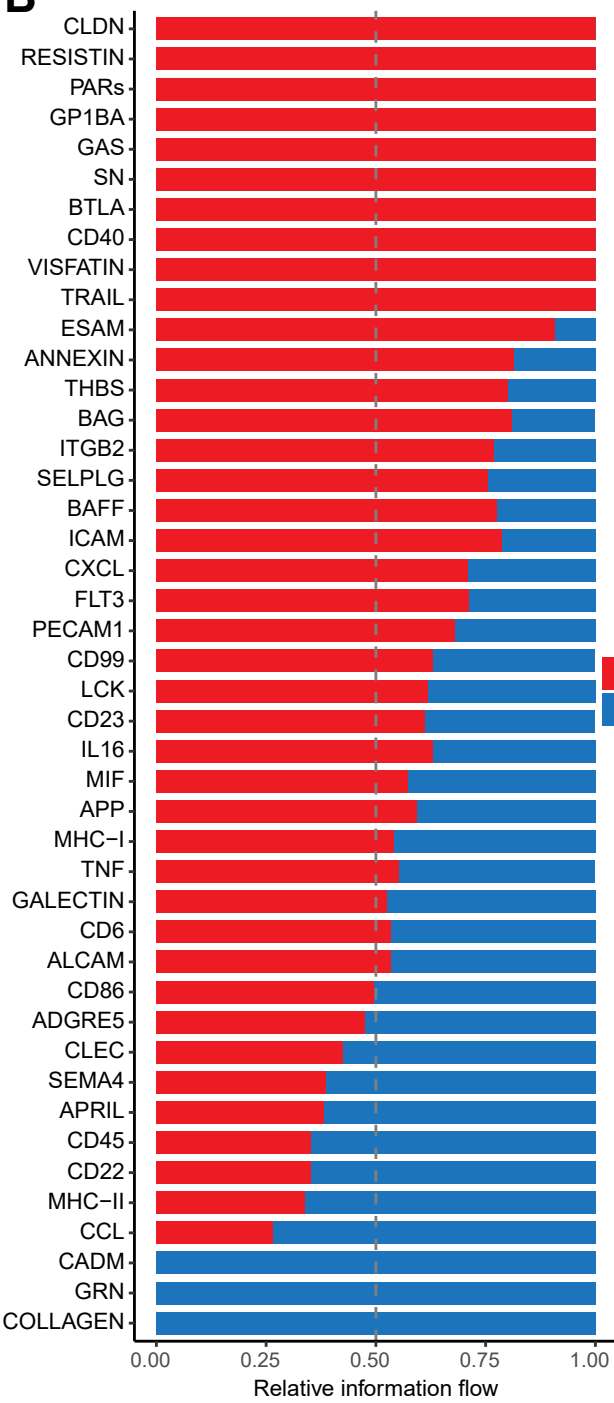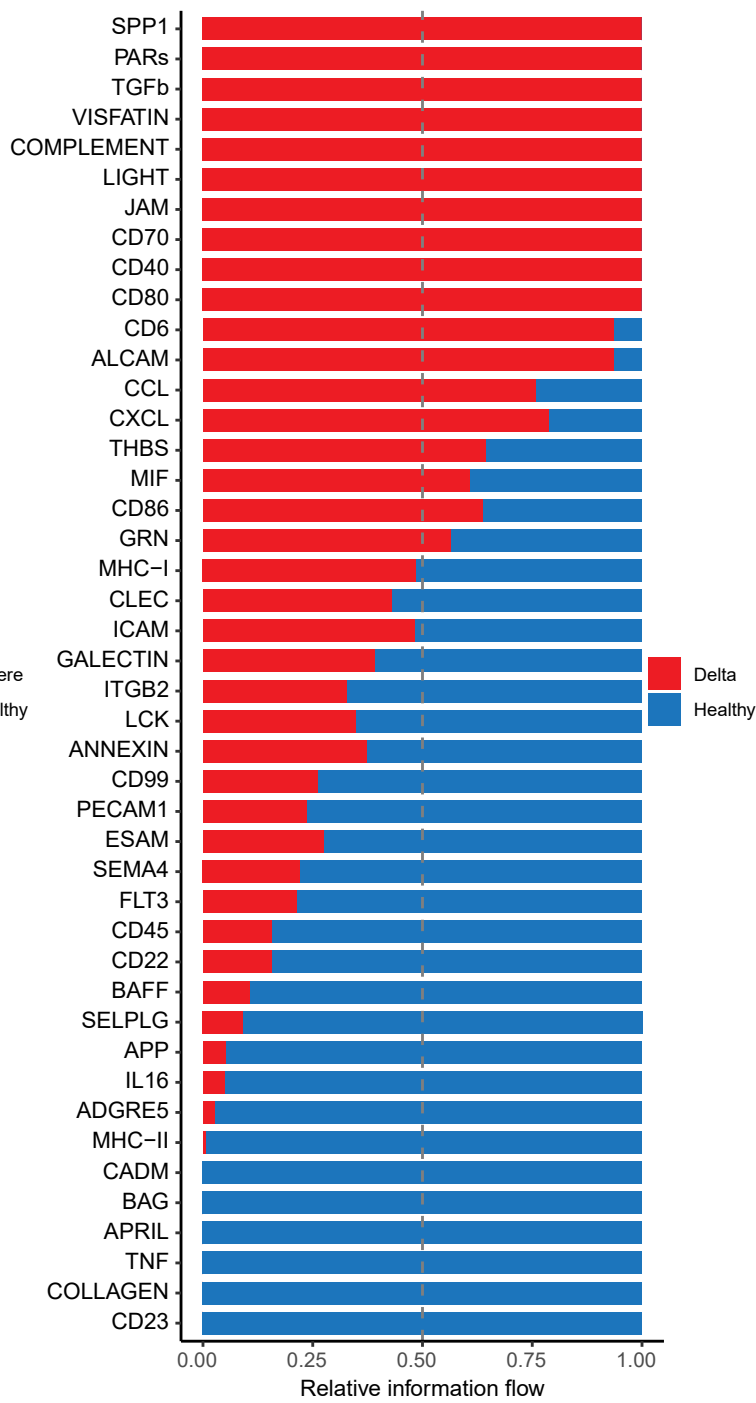

Supplement: Supplementary file 1 [file cells-11-02950-s001.zip › supfiles/S8 copy.pdf]

A

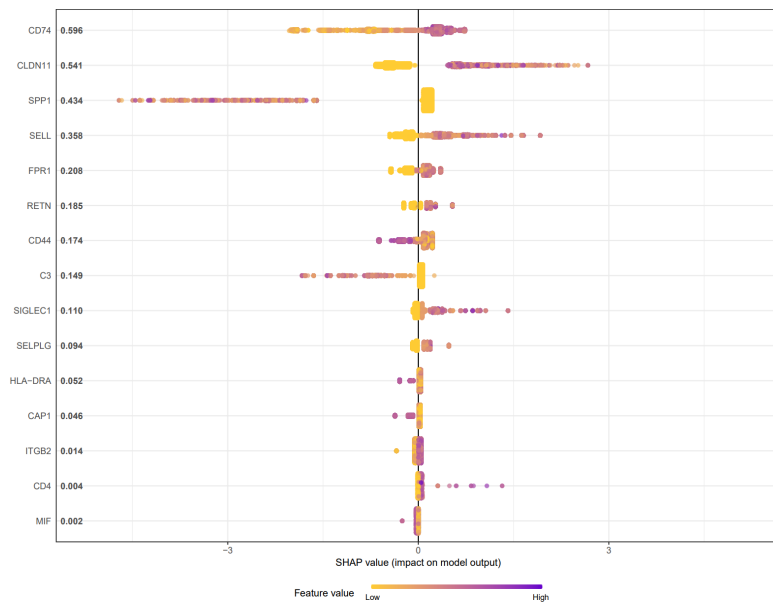

B

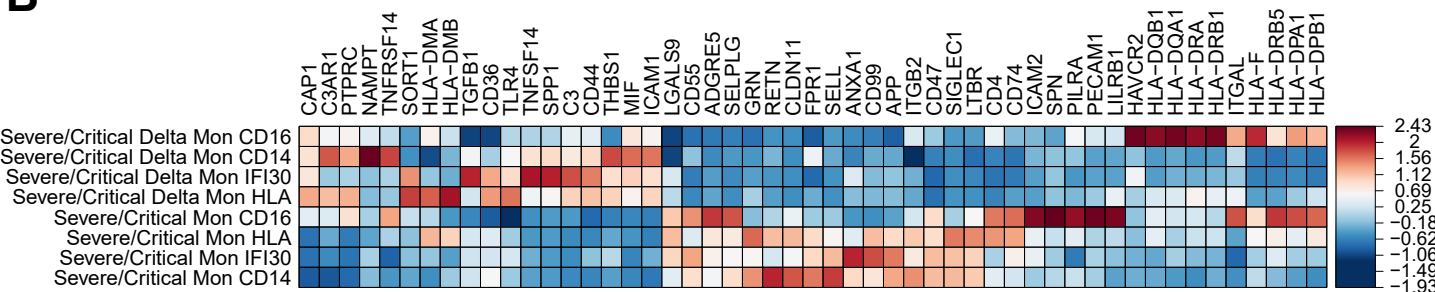

C

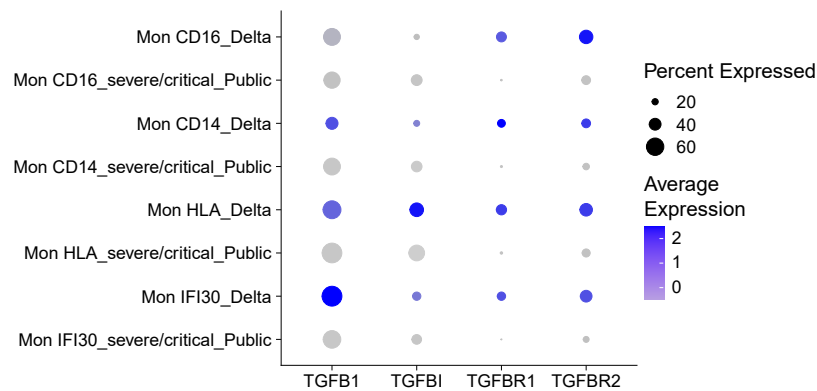

Supplement: Supplementary file 1 [file cells-11-02950-s001.zip › supfiles/S6 copy.pdf]
